# Re-imagining the daniell cell: ampere-hour-level rechargeable Zn–Cu batteries†

**DOI:** 10.1039/d3ee02786d

**Published:** 2023-10-16

**Authors:** Ze He, Jiawei Guo, Fangyu Xiong, Shuangshuang Tan, Yixu Yang, Ruyue Cao, Greta Thompson, Qinyou An, Michael De Volder, Liqiang Mai

**Affiliations:** a State Key Laboratory of Advanced Technology for Materials Synthesis and Processing, Wuhan University of Technology Wuhan 430070 China anqinyou86@whut.edu.cn mlq518@whut.edu.cn; b Institute for Manufacturing, Department of Engineering, University of Cambridge Cambridge CB3 0FS UK mfld2@cam.ac.uk; c College of Materials Science and Engineering, Chongqing University Chongqing 400030 China; d State Key Laboratory of Superlattices and Microstructures, Institute of Semiconductors, Chinese Academy of Sciences Beijing 100083 China; e Hubei Key Laboratory of Electronic Manufacturing and Packaging Integration, Wuhan University Wuhan 430072 China; f Hubei Longzhong Laboratory, Wuhan University of Technology (Xiangyang Demonstration Zone) Xiangyang 441000 Hubei China

## Abstract

The Daniell cell (Cu *vs.* Zn), was invented almost two centuries ago, but has been set aside due to its non-rechargeable nature and limited energy density. However, these cells are exceptionally sustainable because they do not require rare earth elements, are aqueous and easy to recycle. This work addresses key challenges in making Daniell cells relevant to our current energy crisis. First, we propose new approaches to stabilise Zn and Cu plating and stripping processes and create a rechargeable cell. Second, we replace salt bridges with an anion exchange membrane, or a bipolar membrane for alkaline–acid hybrid Zn–Cu batteries operating at 1.56 V. Finally, we apply these changes in pouch cells in order to increase energy and power density. These combined developments result in a rechargeable Daniell cell, which can achieve high areal capacities of 5 mA h cm^−2^ and can easily be implemented in 1 A h pouch cells.

## Abstract 2

The classic Daniell cell was redesigned to make it rechargeable using gel electrolytes, surface coatings, and ion exchange membranes. The proposed cell chemistry is sustainable, straight-forward to recycle, and can be scaled up to 1 A h cells.

## Graphical abstract

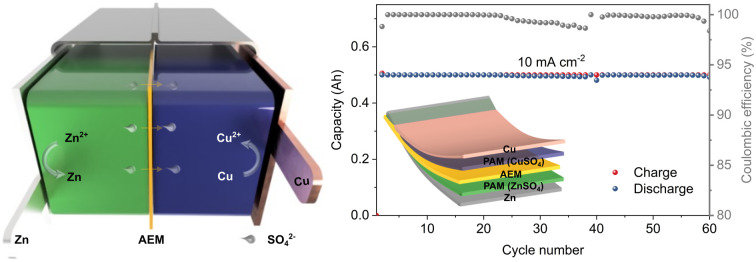

**Broader context**

In the search for ever higher energy densities, battery scientists are revisiting Wittingham's 30-year-old Li metal anodes and are achieving great improvements in energy density as a result. Here, we are leveraging recent developments in electrolyte and separator technologies to revisit the nearly two-centuries-old Cu *vs.* Zn Daniell Cell. We present the first rechargeable Daniell Cell using pouch cell designs and demonstrate its scalability by fabricating 1 A h pouch cells, we further demonstrate the modularity of the concept by changing the anode chemistry to increase the output voltage by 50%. This represents a leap forward in battery sustainability as these cells do not contain any Li, Co, Ni or other scarce or controversial resources, and can in principle be recycled by simply retrieving the Zn and Cu foil. Overall, this proposed modernisation of the two-century-old Daniell cell is particularly timely in our search for new sustainable energy storage solutions to fight climate change.

## Introduction

Metal electrodes are regarded as the “holy grail” of energy storage systems because they intrinsically offer high energy densities, but do not require Co, Ni, or other elements which have geopolitical, toxicological or sustainability issues.^1–4^ Furthermore, metal electrodes simplify the battery manufacturing process: firstly, large-scale manufacturing techniques for thin metal foils are well established; secondly, since metals are excellent electric conductors there is no need to create electrode slurries with conductive additives and binders, which are energy intensive to process and dry. It is therefore unsurprising that S. Witthingham proposed the use of Li metal foils (theoretical capacity of 3861 mA h g^−1^, 2061 mA h cm^−3^) back in the 1970s as anodes for Li-metal batteries (LMBs),^5,6^ and that other metal anodes such as Zn metal foils (theoretical capacity of 820 mA h g^−1^, 5854 mA h cm^−2^) have spurred the development of zinc ion batteries (ZIBs).^7,8^ While impressive advances have been made in cell energy density using metal foil anodes, LMBs and ZIBs still rely on classical cathode materials, many of which present significant sustainability issues related to elements such as Ni, Co, V, *etc.* Moreover, the capacities of these cathode materials often struggle to match those of metal-anodes, leading to cell designs with inefficient negative/positive electrode areal capacity ratios.^9,10^ Finally, with Li resources struggling to keep up with the demand for electric vehicles and grid-scale green energy storage, the need for radically new Li-free battery designs is an increasingly pressing and societally relevant issue.

A solution to the above conundrum was proposed almost two centuries ago by John Frederic Daniell in 1836. The so-called Daniell cell^11^ uses Cu metal (840 mA h g^−1^) as a cathode against the already popular Zn metal anodes (820 mA h g^−1^). In a typical Daniell cell, Zn ions dissolve on the anode side, while Cu^2+^ deposits on the positive side. The charge/discharge reactions of the cell are as follows:At the cathodic electrode: Cu^2+^ + 2e^−^ ↔ Cu *E*^0^ = +0.34 V *vs.* SHEAt the anodic electrode: Zn − 2e^−^ ↔ Zn^2+^ *E*^0^ = −0.76 V *vs.* SHETotal: Cu^2+^ + Zn → Cu + Zn^2+^*E*^0^ = 1.1 V

Under standard conditions, the equilibrium electrochemical potential of the cell is 1.1 V. Importantly, this does not change as a function of the state of charge of the battery, leading to flat voltage plateaus. At the time of their invention, these cells were used in practical applications such as electrical telegraphy. To make the above batteries possible, a salt bridge is needed to separate the electrolytes used on the cathode and anode (catholyte and anolyte), as depicted in Fig. S1 (ESI†). Benefiting from the high capacities of Zn and Cu metal, the Daniell cell delivers a high theoretical energy density of 456 W h kg^−1^ (based on its cathode and anode materials). However, this battery suffers self-discharge and cannot be re-charged due to the severe crossover of Cu^2+^ ions. Additionally, Daniell cells were constructed in beaker cells, which are very bulky and lead to unacceptably low energy and power densities. In recent years, researchers have tried to re-configure Daniell cells in more compact formats, however, this requires solving issues related to metal dendrites piercing through separators and finding suitable alternatives for the salt bridge design.^12^ To address the latter, Li^+^ and Na^+^ ion exchange membranes have been proposed.^13–15^ Xia *et al.* constructed a rechargeable Zn–Cu battery using a ceramic lithium exchange film (LATSP) to separate positive and negative electrolytes and prevent Cu^2+^ crossover.^14^ However, this cell can only deliver a low operating voltage of ∼0.8 V at ∼140 mA g^−1^ due to the high impedance of LATSP. Similarly, Barz *et al.* used a Na^+^ ion exchange membrane as the separator, resulting in a low voltage of ∼0.7 V at a current density of 0.5 mA cm^−2^.^15^ While these proposals are interesting, the use of Li or Na exchange membranes is inadequate because of the observed high overpotentials during cycling and the inevitable introduction of inactive Li or Na salts into the electrolytes, which can significantly reduce the practical energy density of the Zn–Cu batteries. Finally, the stability of Cu plating and stripping is not widely understood and has not been systematically studied yet, which is one of the challenges we focus on in this publication.

Herein, we address the above issues with Daniell cells to make them relevant for 21st century energy storage applications. Firstly, we developed Cu^2+^-based electrolytes and Zn metal coatings to achieve reversible plating/stripping of Cu and Zn to make the Daniell cell reversible. Secondly, we replaced the salt bridge by an anion exchange membrane (AEM), achieving the standard Daniell cell 1.1 V output voltage (see above) with low overpotential. Finally, we replaced the beaker cell design by compact 1 A h pouch cells to increase the volumetric performance of the cells. Furthermore, in a second design, a bipolar membrane (BM) with a hybrid alkaline–acid electrolyte was used to increase the output voltage to 1.56 V. Both batteries showed ultra-stable high operating voltages and excellent cycling stability without the introduction of inactive salts. In the following sections, we will start by discussing the original 1.1 V cell design before moving to high voltage alternatives.

## Results and discussion

### Cu plating/stripping behavior

Our first re-design of the Daniell cell is illustrated in Fig. 3(A). The key change is that rather than using spectator ions delivered from a salt bridge, we propose to use the same electrolyte anions (here SO_4_^2−^) in both half-cells and let these shuttle over an anion exchange membrane. In other words, during discharge, Zn metal strips at the anode and to compensate charge, SO_4_^2−^ shuttles over the AEM to the anode. On the cathode side, the Cu^2+^ is plated which restores charge balance of the lost SO_4_^2−^. While the original Daniell cell was not intended to be rechargeable, we will discuss later on how this process can be reversed on charging by stabilizing the Cu and Zn plating and stripping processes.

**Fig. 1:**
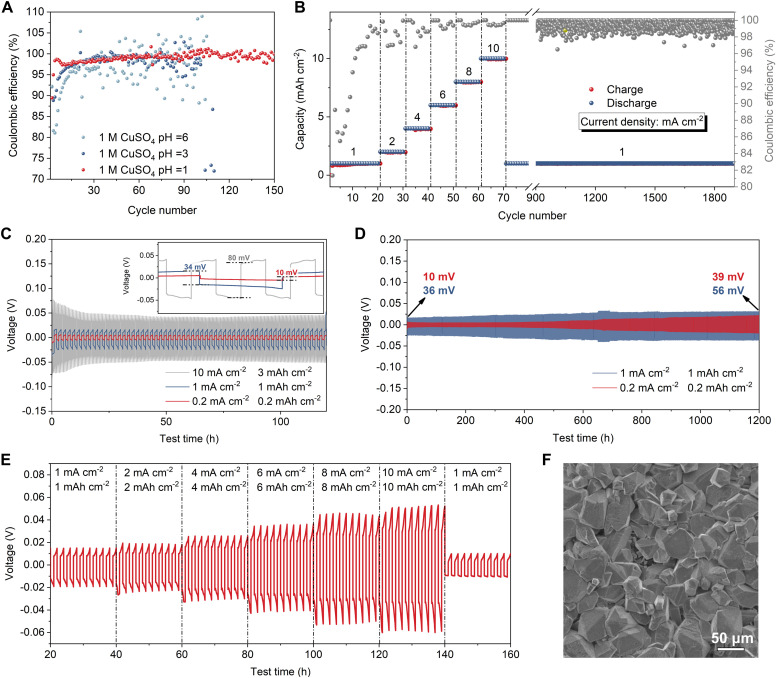
Plating/stripping behavior of Cu metal electrodes. (A) CEs of Cu//CP asymmetrical cell at various electrolyte pH values. (B) CE of Cu//CP asymmetrical cell at current densities from 1 to 10 mA cm^−2^. (C) Time–voltage curves for Cu//Cu symmetrical cells at 0.2, 1 and 10 mA cm^−2^. (D) Long-term cycling performance of Cu//Cu symmetrical cells at 0.2 and 1 mA cm^−2^. (E) Rate performance of Cu//Cu symmetrical cell at current densities from 1 to 10 mA cm^−2^. (F) Scanning electron microscopy (SEM) image of Cu surface after 400 cycles in 1 M CuSO_4_ (pH = 1) electrolyte at 1 mA cm^−2^, 1 mA h cm^−2^.

With the advent of ZIBs, the stabilization of Zn anodes has been well studied,^16,17^ whereas only a handful of researchers have looked into achieving stable stripping and plating of Cu electrodes.^18–20^ We therefore started by systematically studying the reversibility and stability of Cu plating and stripping. Carbon paper was selected as the substrate for the Cu plating/stripping study, owing to its favourable compatibility with Cu, as illustrated in Fig. S2–S5 (ESI†). Cu metal electrodes suffer from unsatisfactory reversibility in neutral electrolytes, while a lower electrolyte pH shows improved Coulombic efficiency (CE) for Cu plating/stripping (Fig. 1(A)). By adjusting the pH of the CuSO_4_ electrolyte to 1, the Cu//CP cell delivers high CEs at various current densities (Fig. 1(B)). The CE of Cu//CP cell reaches 99.3% within 17 cycles at 1 mA cm^−2^, 1 mA h cm^−2^, and remains at ∼99.5% under various test conditions (from 1 mA cm^−2^, 1 mA h cm^−2^ to 10 mA cm^−2^, 10 mA h cm^−2^). When the current density returns to 1 mA cm^−2^, the Cu//CP cell remains stable for more than 1900 h with a high CE of ∼100%. Moreover, the Cu//CP cell exhibits a high CE of 99.7% at a high current density of 10 mA cm^−2^ with a practical areal capacity of 3 mA h cm^−2^, and was stable for more than 400 cycles (Fig. S6, ESI†). This demonstrates excellent reversibility and stability of Cu plating/stripping at industrially relevant areal capacities and current densities. Furthermore, we found that the salt concentration plays an important role in the overpotential of Cu plating/stripping.^21,22^ CuSO_4_ solutions (pH = 1) with concentrations ranging from 0.2 M to 1 M were tested in both Cu//Cu symmetrical cells and Cu//CP asymmetrical cells. These tests revealed that high CuSO_4_ concentration in electrolytes results in a lower overpotential and better reactivity (see CV and EIS data in Fig. S7–S9, ESI†). Interestingly, we found that Cu overpotentials in our optimized electrolyte are much lower than other metal electrodes in aqueous electrolytes (10 mV, 34 mV, and 80 mV at 0.2 mA cm^−2^, 1 mA cm^−2^, and 10 mA cm^−2^, respectively, see Fig. 1(C) and Table S1, ESI†). The long-term cycling tests (Fig. 1(D)) of the symmetrical cells at 0.2 mA cm^−2^, 0.2 mA h cm^−2^ and 1 mA cm^−2^, 1 mA h cm^−2^ confirm the outstanding cycling stability of Cu metal in this electrolyte. The symmetrical cells deliver a long lifetime of more than 1200 h, and over this time, the overpotential increased by only 20 mV (from 36 mV to 56 mV) at 1 mA cm^−2^ and 29 mV (from 10 mV to 39 mV) at 0.2 mA cm^−2^. Moreover, the Cu//Cu symmetrical cell delivers an excellent rate performance with small voltage hysteresis and without soft short circuit^23^ at elevated current density and capacity from 1 mA cm^−2^, 1 mA h cm^−2^ to 10 mA cm^−2^, 10 mA h cm^−2^ (Fig. 1(E) and Fig. S10, ESI†). Fig. 1(F) and Fig. S11 (ESI†) show that little or no dendritic Cu is formed on our electrodes after 400 plating/stripping cycles (1 M CuSO_4_ solution pH = 1 at 1 mA cm^−2^, 1 mA h cm^−2^), which helps explain the excellent long-term cycling performance reported in this work.

### Cu plating/stripping characterization

**Fig. 2:**
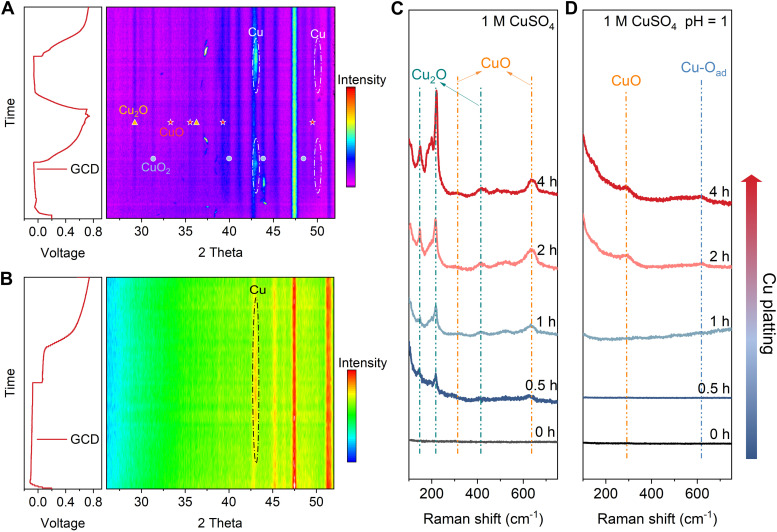
Characterization of copper oxides formation during plating and stripping in different electrolytes. (A) *In situ* XRD test of the CP substrate in 1 M CuSO_4_ electrolyte, and (B) in 1 M CuSO_4_ electrolyte at pH = 1. (C) *Ex situ* Raman spectra of the CP substrate during Cu plating in 1 M CuSO_4_ electrolyte, and (D) in 1 M CuSO_4_ electrolyte at pH = 1, the plating current density is 2 mA cm^−2^.

*In situ* XRD and *ex situ* Raman tests were conducted to better understand the effect of the electrolyte on Cu's cycling stability. XRD diffraction peaks at approximately 43° and 50.1° correspond to the (111) and (200) lattice planes of Cu metal (96-151-2505), while the peaks at approximately 29.5° and 36.4° are attributed to the (011) and (111) lattice planes of Cu_2_O (96-900-5770). Additionally, peaks at around 32.7°, 35.6°, 39.3°, and 49.1° are assigned to the (110), (002), (200), and (20−2) lattice planes of CuO (96-101-1149), respectively. Finally, the peaks at around 31.4°, 39.8°, 43.5°, and 48.7° are identified as the (110), (130), (131), and (042) lattice planes of CuO_2_ (96-901-1548). During the discharge and charge cycles of the Cu//CP asymmetrical cell in 1 M CuSO_4_, peaks at 43° and 50° appear and disappear on the CP electrode, suggesting reversible plating and stripping of Cu (Fig. 2(A)). However, the persistent appearance of peaks at 29.5°, 36.4°, 32.7°, 35.6°, 39.3°, and 49.1° after the first discharge and throughout the subsequent cycles in the 1 M CuSO_4_ electrolyte indicates the irreversible formation of CuO_*x*_ (Cu_2_O, CuO, and CuO_2_) during Cu plating in a neutral electrolyte, which may contribute to the reduction in CE discussed above. In contrast, Fig. 2(B) shows reversible Cu plating and stripping without any observable CuO_*x*_ formation in 1 M CuSO_4_ electrolyte at pH = 1. The *ex situ* Raman spectra in Fig. 2(C) and (D) provide additional evidence of suppression of CuO_*x*_ (peaks at 150 cm^−1^, 220 cm^−1^, and 415 cm^−1^ are assigned to Cu_2_O,^24–26^ while the peaks at 303 cm^−1^ and 636 cm^−1^ are identified as CuO^27^) formation in the acidic electrolyte. Moreover, XRD and Raman measurements of cycled Cu in different electrolytes further confirm the suppression of CuO_*x*_ formation in 1 M CuSO_4_ (pH = 1) electrolyte during cycling (Fig. S12 and S13, ESI†).

### Zn metal anode modification

In a similar manner to Cu cathodes, the development of stable dendritic-free Zn anodes is key to achieving rechargeable pouch cells, which are much more sensitive to dendrite-induced short circuits than mold and beaker cell designs. In this work, we stabilize the Zn plating and stripping by coating a 1 μm Sn layer on the Zn metal anode (see Fig. S14–S16, ESI†). These Sn-coated Zn anodes show better compatibility with the ZnSO_4_ electrolyte than the bare Zn anode (Fig. S17 and S18, ESI†). Density functional theory (DFT) calculations in Fig. S19 (ESI†) show the good Zn affinity of Sn, which corroborates how Sn might help regulate Zn plating. This result is also consistent with recent studies on Zn–ion batteries.^28–30^ Compared to a bare Zn anode, the Sn-coated electrodes show a 6-fold increase in cycle life, with a lifetime of more than 1200 h at industrially relevant current densities (1 mA cm^−2^ and 10 mA cm^−2^, see Fig. S20, ESI†). Additionally, the Sn-coated electrodes exhibit much lower overpotentials at various current densities (29, 99, 157 mV at 1, 15, 30 mA cm^−2^, respectively) during plating/stripping than the bare Zn anode (see Fig. S21, ESI†). Combined with the low overpotentials previously observed for Cu cathodes, this offers good prospects for high energy efficiency Zn–Cu batteries. It is worth mentioning that other coatings for Zn anodes have been proposed in literature such as CaCO_3_,^31^ MXene,^32^ and kaolin,^33^ however, in this work we propose Sn layers because of the ease of Sn deposition and its low Zn plating overpotential.

### H-type mold cells assembling

**Fig. 3:**
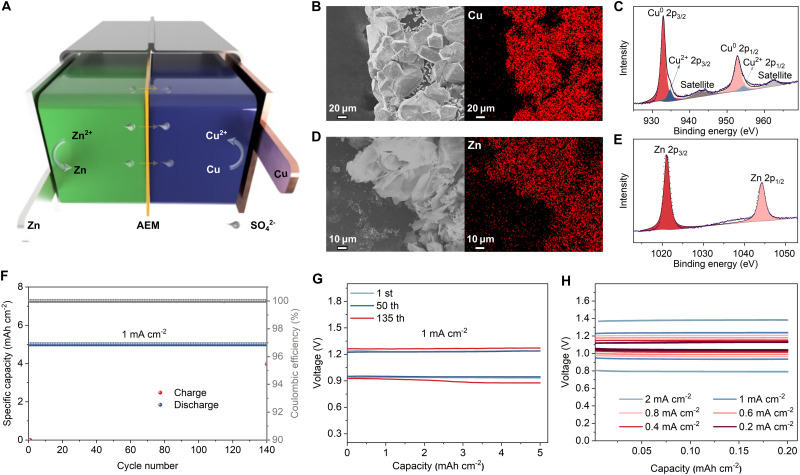
Reaction mechanism of the AEM Zn–Cu battery and its electrochemical performance in H-type mold cells. (A) Schematic diagram of the AEM Zn–Cu battery. (B)–(D) EDS elemental mappings of Cu (B) and Zn (D) for CP after being discharged in a cathode-free cell and charged in an anode-free cell respectively. XPS spectra of Cu 2p (C) and Zn 2p (E) for CP after being discharged in a cathode-free cell and charged in an anode-free cell respectively. (F) Cycling performance of the AEM Zn–Cu cell. (G) GCD curves of the AEM Zn–Cu cell at 1 mA cm^−2^ for the 1st, 50th, and 135th cycles. (H) Rate performance of the AEM Zn–Cu cell.

After testing symmetric cells, we constructed H-type mold half cells composed of only the Cu cathode or Zn anode *versus* CP (see Fig. S22, ESI†), which confirmed that the individual anode and cathode reactions are plating/stripping of each metal. SEM images and energy dispersive X-ray spectroscopy (EDS) elemental mapping of CP after discharge in the cathode-free cell, using 1 M CuSO_4_ electrolyte (pH = 1), clearly show plated Cu particles (Fig. 3(B)) with a regular shape and smooth surface morphology (Fig. S23, ESI†). The X-ray photoelectron spectroscopy (XPS) spectrum (Fig. 3(C)) displays the typical peaks of Cu^0^ 2p_3/2_ and Cu^0^ 2p_1/2_ at 932.9 eV and 952.7 eV, respectively.^34,35^ The presence of weak peaks at 934.7 eV and 954.3 eV and their satellite peaks demonstrate the existence of trace amounts of Cu^2+^,^36–38^ which is likely due to the formation of a small amount of CuO_*x*_ during the Cu plating. In comparison, deposited Cu in the neutral 1 M CuSO_4_ electrolyte possesses a rough surface morphology (Fig. S24, ESI†) and significantly stronger peaks at 934.9 eV and 954.6 eV in the XPS spectrum (Fig. S25, ESI†), indicating that irreversible CuO_*x*_ formation is substantially inhibited in the modified electrolyte. These findings provide additional insight into our previous measurements in coin cells, which demonstrated improved CE at lower electrolyte pH. Similarly, SEM and EDS elemental mapping of the CP after charging in the anode-free cell show deposited Zn metal (Fig. 3(D)). The XPS Zn 2p spectrum of the charged CP shows two distinct peaks located at 1021.1 eV and 1044.2 eV, corresponding to the Zn 2p_3/2_ and Zn 2p_1/2_, respectively^39–41^ (Fig. 3(E)). These results verify the bilateral plating/stripping reaction mechanism of the AEM aqueous Zn–Cu metal battery.

In order to test the full Zn–Cu cell, H-type mold cells were initially assembled. The optimized CuSO_4_ electrolyte was used on the cathode side, along with the improved Sn-coated Zn anodes (see Fig. S26, ESI†). These Zn–Cu batteries showed significantly improved electrochemical performance compared to standard AEM Zn–Cu cells, including higher CE and better cycling stability (Fig. S27, ESI†). The specific capacity of the Cu metal cathode was verified based on a pre-defined amount of Cu stripped, at a current density of 200 mA g^−1^, the theoretical capacity of ∼840 mA h g^−1^ was obtained (Fig. S28, ESI†). Notably, this specific capacity is significantly higher than that of other cathode materials, such as manganese oxides,^42–44^ vanadium oxides,^7,45,46^ polyanionic compounds,^47,48^ Prussian blue analogues,^49–51^ and organic compounds,^52–54^ that are commonly used in battery systems with a Zn metal anode. Our cells demonstrated stable cycling for more than 1400 h (140 cycles) with a high areal capacity of 5 mA h cm^−2^ at 1 mA cm^−2^ (Fig. 3(F)). The galvanostatic charge–discharge (GCD) curves display an ultra-stable charge/discharge plateau during cycling (Fig. 3(G)). The discharge voltage decreased only 0.04 V after 135 cycles (from 0.94 V to 0.9 V), indicating excellent stability. In addition, the cells can also be cycled at ultra-high areal capacities of 10 mA h cm^−2^ and 20 mA h cm^−2^ for 30 and 10 cycles, respectively (Fig. S29 and S30, ESI†). Improving the cycling stability of high areal capacity electrodes is important as it reduces the amount of AEM used in the cells, which is the most expensive part of these batteries.

However, there was a noticeable discharge voltage decrease (from 1.05 V to 0.80 V) when the current density increased from 0.2 to 2 mA cm^−2^, and the cells failed to run under current densities higher than 3 mA cm^−2^ (Fig. 3(H) and Fig. S31, ESI†). This is attributed to the relatively high overpotential, which is itself due to the long ion diffusion distance in mold cells.

### Zn–Cu pouch cells construction

**Fig. 4:**
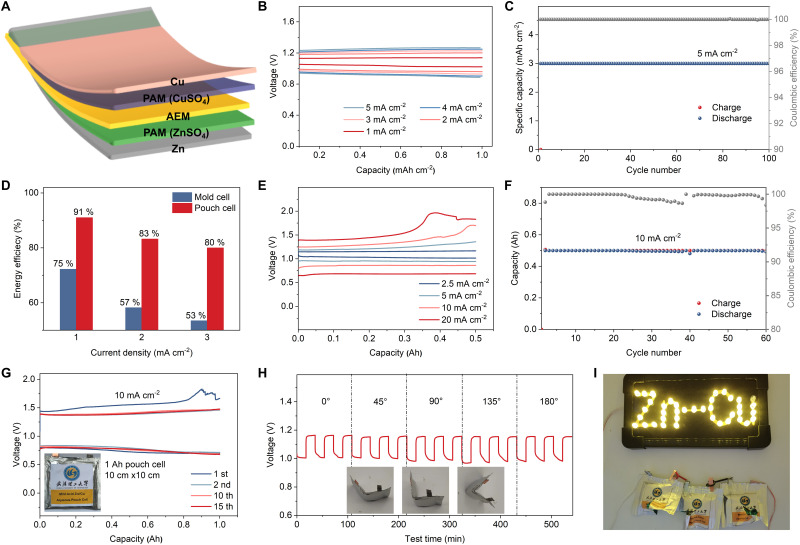
Electrochemical and safety performance of the AEM Zn–Cu pouch cells. (A) Schematic diagram of the AEM Zn–Cu pouch cell. (B) Rate performance of the AEM Zn–Cu pouch cell at current densities from 1 to 5 mA cm^−2^. (C) Cycling performance of the AEM Zn–Cu pouch cell at 5 mA cm^−2^. (D) Energy efficiencies of the mold cells and pouch cells at various current densities. (E) Rate performance of 0.5 A h AEM Zn–Cu pouch cell at current densities from 2.5 to 20 mA cm^−2^. (F) Cycling performance of 0.5 A h AEM Zn–Cu pouch cell at high current density of 10 mA cm^−2^. (G) GCD curves of 1 A h AEM Zn–Cu pouch cell at a high current density of 10 mA cm^−2^. Inset is a digital photo of the pouch cell. (H) Time–voltage curves of the AEM Zn–Cu pouch cell under different angles for bending. (I) LEDs lit by a series of three AEM Zn–Cu pouch cells after being cut.

The experiments above are all carried out in coin cells or H-cells to optimize the electrolytes and achieve highly reversible metal electrodes, both of which are key to implementing our modified AEM Daniell cells in a pouch format. An additional challenge when moving to pouch cells is that Daniell cells require a buffer of Cu and Zn ions in each half cell. In this work, we overcame this issue by using polyacrylamide (PAM)/CuSO_4_ gel catholyte and PAM/ZnSO_4_ gel anolyte, as illustrated in Fig. 4(A). Due to the high ionic conductivity (1.2 × 10^−1^ S cm^−1^) of PAM^55^ and the decreased distance between cathode and anode in a pouch cell compared to an H-type cell, there is reduced internal resistance and thus a much lower overpotential and improved rate performance are observed. The assembled pouch cell delivered discharge voltages of 1.05, 0.99, 0.97, 0.95, and 0.93 V at current densities of 1, 2, 3, 4, and 5 mA cm^−2^, respectively (Fig. 4(B)). When cycled at a current density of 1.5 mA cm^−2^ with an areal capacity of 3 mA h cm^−2^, the pouch cell exhibited excellent cycling stability with a slight overpotential increase (from 0.08 to 0.18 V) after 100 cycles (Fig. S32, ESI†). The discharge voltage decreases from 1.04 V to 0.95 V over 200 cycles (Fig. S33, ESI†). A contribution to the increase in impedance could be due to the formation of CuO_*x*_, previously observed as small Cu^2+^ peaks in XPS (Fig. 3(C)). Surprisingly, even at a very high current density of 5 mA cm^−2^, the cell was able to cycle more than 100 times at a capacity of 3 mA h cm^−2^ (Fig. 4(C)). The corresponding GCD curves (Fig. S34, ESI†) and discharge voltage (Fig. S35, ESI†) show a voltage drop of 54 mV over 150 cycles.

The pouch cells demonstrated a notable improvement in energy efficiency compared to the mold cells. As shown in Fig. 4(D), our pouch cells deliver a 16% improvement in energy efficiency at 1 mA cm^−2^, and a 27% improvement at 3 mA cm^−2^, which is a further illustration of why changing the format of Daniell cells to pouch cells is important. Moreover, our pouch cell design mitigates the crossover of Cu^2+^, which contributed to the good cycling stability and suppressed self-discharge (Fig. S36 and S37, ESI†). The cathode-free AEM Zn–Cu pouch cell delivered an average CE of 98.4%, which further confirms the excellent cycling performance of our pouch cell design (Fig. S38, ESI†).

We anticipate that the achieved excellent cycling stabilities at commercially relevant loadings are achieved by a combination of electrolyte optimization, our Sn-coated Zn anode, and the AEM and PAM gel electrolytes contributing to a uniform ion flux distribution. We evaluated the rate and cycling performance of large format AEM Zn–Cu pouch cells (0.5 A h) with a thin Cu cathode (thickness: 15 μm). The GCD curves of the 0.5 A h pouch cell at various current densities demonstrate its outstanding rate performance, with operating voltages of 1.02, 0.95, and 0.86 V achieved at current densities of 2.5, 5, and 10 mA cm^−2^, respectively (Fig. 4(E)). Even at an ultra-high current density of 20 mA cm^−2^, the high-capacity pouch cell delivered a decent operating voltage of 0.68 V, corresponding to a high power density of 1017.6 W kg_Cu_^−1^. The 0.5 A h pouch cell can stably run for more than 60 cycles at 10 mA cm^−2^ (Fig. 4(F)), corresponding to the high areal capacity of 10 mA h cm^−2^ and energy density of 598 W h kg^−1^ (based on the Cu metal cathode). Moreover, we further assembled 1 A h pouch cells to verify that the cell manufacturing process can be easily scaled up. The 1 A h cells demonstrate similar GCD curves as low-capacity cells at a low current density of 0.5 mA cm^−2^ (Fig. S39, ESI†) and can stably run for more than 15 cycles at a high current density of 10 mA cm^−2^ (Fig. 4(G)). Finally, an important advantage of the proposed cells is that they do not rely on coated multi-component electrodes, and as a result there are no issues with delamination upon extreme bending. This means that our pouch cells exhibit almost unchanged charge/discharge curves at different bending angles (Fig. 4(H)). More impressively, the cells do not use flammable electrolytes and are not moisture sensitive; furthermore, we found that they maintain stable operation when punctured (Fig. S40, ESI†) or cut (Fig. 4(I)). We are therefore confident that our AEM Zn–Cu cells offer safety and flexibility benefits in addition to their improved sustainability, making them potentially suitable for wearable devices.

### High-voltage Zn–Cu cell design

**Fig. 5:**
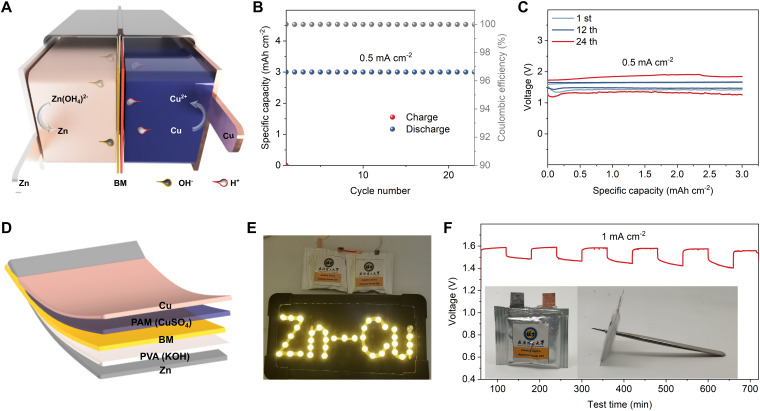
Construction and performance of the alkaline–acid hybrid Zn–Cu batteries. (A) Schematic diagram of the alkaline–acid hybrid Zn–Cu battery. (B and C) Cycling performance of the alkaline–acid hybrid Zn–Cu cell at 0.5 mA cm^−2^ (B) and the corresponding GCD curves for the 1st, 12th, and 24th cycles (C). (D) Schematic illustration of the alkaline–acid hybrid Zn–Cu pouch cell. (E) Two alkaline–acid hybrid Zn–Cu pouch cells in series lighting up LEDs. (F) Time–voltage curves of the alkaline–acid hybrid Zn–Cu pouch cell after being punctured. Inset is a photo of the punctured cell.

An intrinsic limitation of the Daniell cell is its nominal voltage of 1.1 V, which is set by the combination of Cu and Zn electrodes. To increase the output voltage, and to demonstrate the versatility of our modified cell design, we introduce a second set of changes to the Daniell cell: namely an alkaline Zn redox reaction on the anode of the cell (Fig. 5(A)). This alkaline–acid hybrid battery replaces the zinc sulfate anode electrolyte with 6 M KOH + 0.3 M Zn(Ac)_2_ solution, and switches the AEM with a BM. The BM only allows H^+^ and OH^−^ to penetrate through the cation exchange layer and anion exchange layer respectively, preventing the neutralization of the cathodic and anodic electrolytes (the mechanism is detailed in Fig. S41 and Table S2, ESI†).^56,57^ The electrochemical reactions of an alkaline–acid hybrid Zn–Cu battery during the charge/discharge process are summarized as follows:At the positive electrode: Cu^2+^ + 2e^−^ ↔ Cu *E*^0^ = +0.34 V *vs.* SHEAt the negative electrode: Zn + 4OH^−^ − 2e^−^ ↔ Zn(OH)_4_^2−^ *E*^0^ = −1.22 V *vs.* SHETotal: Cu^2+^ + Zn + 4OH^−^ ↔ Cu + Zn(OH)_4_^2−^ *E* = 1.56 V

The proposed alkaline–acid hybrid Zn–Cu battery possesses a high theoretical voltage of 1.56 V due to the low redox potential of Zn metal in alkaline electrolyte.^42,58–60^ Similar to the previous approach, both H-type cells (Fig. S42, ESI†) and pouch cells were assembled to verify feasibility. The alkaline–acid hybrid Zn–Cu H-type cell maintained a stable capacity of 3 mA h cm^−2^ at 0.5 mA cm^−2^ for 288 h (Fig. 5(B)), exhibiting a high initial discharge voltage of 1.42 V and maintaining 1.36 V after 24 cycles (Fig. 5(C)). We then developed a polyvinyl alcohol (PVA)/KOH gel electrolyte for the anode and paired it with the same PAM/CuSO_4_ gel used on the cathode of AEM Zn–Cu cells (see Fig. 5(D)). The alkaline–acid hybrid Zn–Cu pouch cells delivered an open-circuit voltage of 1.56 V and demonstrated a relatively low self-discharge rate (Fig. S43 and S44, ESI†). Two alkaline–acid hybrid Zn–Cu pouch cells in series were able to light up LEDs as shown in Fig. 5(E). Moreover, the punctured alkaline–acid hybrid Zn–Cu pouch cell still displayed a stable charge/discharge performance (Fig. 5(F)), indicating excellent stability and safety. These cells not only demonstrate opportunities to increase the cell voltage, but also show that the anode and cathode reactions can be modified independently while retaining good stability and compatibility with pouch cell designs.

## Conclusions

In summary, this work has brought the Daniell cell to the 21st century by leveraging recent advances made in the field of metal electrodes, anion exchange membranes, gel electrolytes, and pouch cell designs. The original Daniell cell is very attractive because it does not rely on any rare earth elements or a complex cell design, but it is limited in practicality since it is not rechargeable and relies on bulky beaker cells and salt bridges. We overcame these challenges by using anion exchange membranes with gel-based electrolytes to allow for implementation in pouch cells and re-charging the cell. This re-designed all-metal-electrode battery has numerous advantages including: flat voltage plateaus; excellent safety; bending flexibility; material sustainability; and straight-forward recycling, since no rare earth or toxic materials or electrolytes are used in our designs. Our full-cells achieve stable operation for 140 cycles at a high capacity of 5 mA h cm^−2^ and can operate at ultra-high areal capacities of 10 mA h cm^−2^ and 20 mA h cm^−2^. Stabilizing these high areal capacities is important in the long term as it reduces the amount of AEM needed and therefore reduces the cell cost. A comparison of the cost of cell parts of the proposed batteries and Li ion batteries is provided in Table S3 (ESI†). Moreover, we demonstrate that our design can be scaled up to 1 A h cells and that it is modular. We prove the latter by exchanging the anode electrolyte for an alkaline electrolyte to increase the operating voltage to 1.56 V. With excellent safety merits, outstanding electrochemical performance, and sustainable use of materials, we anticipate that the proposed all-metal Zn–Cu batteries will lay the groundwork for new energy storage systems that are particularly attractive for large-scale renewable energy storage applications.

## Conflicts of interest

There are no conflicts of interest to declare.

## Supplementary Material

EE-016-D3EE02786D-s001

## References

[cit1] Chen S., Zheng J., Mei D., Han K. S., Engelhard M. H., Zhao W., Xu W., Liu J., Zhang J. G. (2018). Adv. Mater..

[cit2] Chen S., Zheng J., Yu L., Ren X., Engelhard M. H., Niu C., Lee H., Xu W., Xiao J., Liu J., Zhang J.-G. (2018). Joule.

[cit3] Wang H., Tan R., Yang Z., Feng Y., Duan X., Ma J. (2021). Adv. Energy Mater..

[cit4] Wang H., Yu D., Kuang C., Cheng L., Li W., Feng X., Zhang Z., Zhang X., Zhang Y. (2019). Chem.

[cit5] Lin D., Liu Y., Cui Y. (2017). Nat. Nanotechnol..

[cit6] Whittingham M. S. (1976). Science.

[cit7] Kundu D., Adams B. D., Duffort V., Vajargah S. H., Nazar L. F. (2016). Nat. Energy.

[cit8] Jia X., Liu C., Neale Z. G., Yang J., Cao G. (2020). Chem. Rev..

[cit9] Liu J., Bao Z., Cui Y., Dufek E. J., Goodenough J. B., Khalifah P., Li Q., Liaw B. Y., Liu P., Manthiram A., Meng Y. S., Subramanian V. R., Toney M. F., Viswanathan V. V., Whittingham M. S., Xiao J., Xu W., Yang J., Yang X.-Q., Zhang J.-G. (2019). Nat. Energy.

[cit10] Kwon M., Lee J., Ko S., Lim G., Yu S.-H., Hong J., Lee M. (2022). Energy Environ. Sci..

[cit11] Boulabiar A., Bouraoui K., Chastrette M., Abderrabba M. (2004). J. Chem. Educ..

[cit12] Xu C., Lei C., Li J., He X., Jiang P., Wang H., Liu T., Liang X. (2023). Nat. Commun..

[cit13] Zhang H., Yang T., Wu X., Zhou Y., Yang C., Zhu T., Dong R. (2015). Chem. Commun..

[cit14] Dong X., Wang Y., Xia Y. (2014). Sci. Rep..

[cit15] Jameson A., Khazaeli A., Barz D. P. J. (2020). J. Power Sources.

[cit16] Yuan L., Hao J., Kao C.-C., Wu C., Liu H.-K., Dou S.-X., Qiao S.-Z. (2021). Energy Environ. Sci..

[cit17] Zheng J., Archer L. A. (2021). Sci. Adv..

[cit18] Liang G., Mo F., Yang Q., Huang Z., Li X., Wang D., Liu Z., Li H., Zhang Q., Zhi C. (2019). Adv. Mater..

[cit19] Yu M., Sui Y., Sandstrom S. K., Wu C. Y., Yang H., Stickle W., Luo W., Ji X. (2022). Angew. Chem., Int. Ed..

[cit20] Song X., Wang C., Wang D., Peng H., Wang C., Wang C., Fan W., Yang J., Qian Y. (2023). J. Energy Chem..

[cit21] Li S., Jiang M., Xie Y., Xu H., Jia J., Li J. (2018). Adv. Mater..

[cit22] Dong N., Zhang F., Pan H. (2022). Chem. Sci..

[cit23] Li Q., Chen A., Wang D., Pei Z., Zhi C. (2022). Joule.

[cit24] Chan H. Y. H., Takoudis C. G., Weaver M. J. (1999). J. Phys. Chem. B.

[cit25] Yang P. P., Zhang X. L., Gao F. Y., Zheng Y. R., Niu Z. Z., Yu X., Liu R., Wu Z. Z., Qin S., Chi L. P., Duan Y., Ma T., Zheng X. S., Zhu J. F., Wang H. J., Gao M. R., Yu S. H. (2020). J. Am. Chem. Soc..

[cit26] Zhang W., Huang C., Xiao Q., Yu L., Shuai L., An P., Zhang J., Qiu M., Ren Z., Yu Y. (2020). J. Am. Chem. Soc..

[cit27] Deng Y., Handoko A. D., Du Y., Xi S., Yeo B. S. (2016). ACS Catal..

[cit28] Wang L., Huang W., Guo W., Guo Z. H., Chang C., Gao L., Pu X. (2022). Adv. Funct. Mater..

[cit29] Xiong P., Kang Y., Yuan H., Liu Q., Baek S. H., Park J. M., Dou Q., Han X., Jang W.-S., Kwon S. J., Kim Y.-M., Li W., Park H. S. (2022). Appl. Phys. Rev..

[cit30] Zhai S., Shi X., Jiang K., Tan X., Zhang W., Zhang J., Zhang H., Li Z. (2022). Chem. Eng. J..

[cit31] Kang L., Cui M., Jiang F., Gao Y., Luo H., Liu J., Liang W., Zhi C. (2018). Adv. Energy Mater..

[cit32] Zhang N., Huang S., Yuan Z., Zhu J., Zhao Z., Niu Z. (2021). Angew. Chem., Int. Ed..

[cit33] Deng C., Xie X., Han J., Tang Y., Gao J., Liu C., Shi X., Zhou J., Liang S. (2020). Adv. Funct. Mater..

[cit34] Lee D., Sun S., Kwon J., Park H., Jang M., Park E., Son B., Jung Y., Song T., Paik U. (2020). Adv. Mater..

[cit35] Peng J., Chen B., Wang Z., Guo J., Wu B., Hao S., Zhang Q., Gu L., Zhou Q., Liu Z., Hong S., You S., Fu A., Shi Z., Xie H., Cao D., Lin C. J., Fu G., Zheng L. S., Jiang Y., Zheng N. (2020). Nature.

[cit36] Liu L., Li W., Xiong Z., Xia D., Yang C., Wang W., Sun Y. (2019). J. Hazard. Mater..

[cit37] Speckmann H., Haupt S., Strehblow H. H. (1988). Surf. Interface Anal..

[cit38] Poulston S., Parlett P., Stone P., Bowker M. (1996). Surf. Interface Anal..

[cit39] Biesinger M. C., Lau L. W. M., Gerson A. R., Smart R. S. C. (2010). Appl. Surf. Sci..

[cit40] Ma L., Li Q., Ying Y., Ma F., Chen S., Li Y., Huang H., Zhi C. (2021). Adv. Mater..

[cit41] Hao J., Li B., Li X., Zeng X., Zhang S., Yang F., Liu S., Li D., Wu C., Guo Z. (2020). Adv. Mater..

[cit42] Pan H., Shao Y., Yan P., Cheng Y., Han K. S., Nie Z., Wang C., Yang J., Li X., Bhattacharya P., Mueller K. T., Liu J. (2016). Nat. Energy.

[cit43] Zhang N., Cheng F., Liu J., Wang L., Long X., Liu X., Li F., Chen J. (2017). Nat. Commun..

[cit44] Alfaruqi M. H., Mathew V., Gim J., Kim S., Song J., Baboo J. P., Choi S. H., Kim J. (2015). Chem. Mater..

[cit45] Xia C., Guo J., Li P., Zhang X., Alshareef H. N. (2018). Angew. Chem., Int. Ed..

[cit46] Ming F., Liang H., Lei Y., Kandambeth S., Eddaoudi M., Alshareef H. N. (2018). ACS Energy Lett..

[cit47] Li G., Yang Z., Jiang Y., Jin C., Huang W., Ding X., Huang Y. (2016). Nano Energy.

[cit48] Wang F., Hu E., Sun W., Gao T., Ji X., Fan X., Han F., Yang X.-Q., Xu K., Wang C. (2018). Energy Environ. Sci..

[cit49] Yang Q., Mo F., Liu Z., Ma L., Li X., Fang D., Chen S., Zhang S., Zhi C. (2019). Adv. Mater..

[cit50] Ma L., Chen S., Long C., Li X., Zhao Y., Liu Z., Huang Z., Dong B., Zapien J. A., Zhi C. (2019). Adv. Energy Mater..

[cit51] Zhang L., Chen L., Zhou X., Liu Z. (2015). Adv. Energy Mater..

[cit52] Nam K. W., Kim H., Beldjoudi Y., Kwon T. W., Kim D. J., Stoddart J. F. (2020). J. Am. Chem. Soc..

[cit53] Shi H. Y., Ye Y. J., Liu K., Song Y., Sun X. (2018). Angew. Chem., Int. Ed..

[cit54] Kundu D., Oberholzer P., Glaros C., Bouzid A., Tervoort E., Pasquarello A., Niederberger M. (2018). Chem. Mater..

[cit55] Dong H., Li J., Guo J., Lai F., Zhao F., Jiao Y., Brett D. J. L., Liu T., He G., Parkin I. P. (2021). Adv. Mater..

[cit56] Chao D., Ye C., Xie F., Zhou W., Zhang Q., Gu Q., Davey K., Gu L., Qiao S. Z. (2020). Adv. Mater..

[cit57] Dai Y., Li J., Chen L., Le K., Cai Z., An Q., Zhang L., Mai L. (2021). ACS Energy Lett..

[cit58] Zhong C., Liu B., Ding J., Liu X., Zhong Y., Li Y., Sun C., Han X., Deng Y., Zhao N., Hu W. (2020). Nat. Energy.

[cit59] Hao J., Li X., Zeng X., Li D., Mao J., Guo Z. (2020). Energy Environ. Sci..

[cit60] Yuan X., Wu X., Zeng X. X., Wang F., Wang J., Zhu Y., Fu L., Wu Y., Duan X. (2020). Adv. Energy Mater..

