# Supplementary material for: Re-imagining the daniell cell: ampere-hour-level rechargeable Zn–Cu batteries
Source: Energy Environ Sci. 2023 Oct 16;16(12):5832–41. doi: 10.1039/d3ee02786d (PMC10698845; doi:10.1039/d3ee02786d)
Supplement: EE-016-D3EE02786D-s001 [file EE-016-D3EE02786D-s001.pdf]

Electronic Supplementary Material (ESI) for Energy & Environmental Science.
This journal is © The Royal Society of Chemistry 2023

Supplemental information

Re-imagining the Daniell cell:

Ampere-hour-level rechargeable Zn-Cu batteries

Ze He,[1,2] Jiawei Guo,[1] Fangyu Xiong,[1] Shuangshuang Tan,[3] Yixu Yang,[1] Ruyue Cao,[4,5] Greta Thompson,[2] Qinyou An,[1,6]* Michael De Volder,[2]* Liqiang Mai[1,6]*

[1]Key Laboratory of Advanced Technology for Materials Synthesis and Processing, Wuhan University of Technology, Wuhan, 430070, China

[2]Institute for Manufacturing, Department of Engineering, University of Cambridge, Cambridge CB3 0FS, UK

[3]College of Materials Science and Engineering, Chongqing University, Chongqing 400030, China

[4]State Key Laboratory of Superlattices and Microstructures, Institute of Semiconductors, Chinese Academy of Sciences, Beijing 100083, China

[5]Department of Engineering, University of Cambridge, Cambridge, CB2 1PZ, UK

[6]Hubei Longzhong Laboratory, Wuhan University of Technology (Xiangyang Demonstration Zone), Xiangyang 441000, Hubei, China

* Corresponding author (email: anqinyou86@whut.edu.cn; mfld2@cam.ac.uk; mlq518@whut.edu.cn)

These authors contributed equally: Ze He, Jiawei Guo.

1. Experimental Section

Preparation of the aqueous electrolytes

The cathodic electrolyte consisted of 1.0 M $CuSO_4$ in de-ionized (DI) water, and the pH was adjusted to 1 by the addition of H_2SO_4. The anodic electrolyte for the AEM Zn-Cu battery consisted of 2 M $ZnSO_4$ in DI water; the anodic electrolyte for the alkaline-acid hybrid Zn-Cu battery consisted of 6 M KOH + 0.3 M $Zn(Ac)_2$ in DI water.

Preparation of the Sn-coated Zn anode

The commercial Zn foil (100 μm) was polished with 2000 mesh sandpaper and washed with ethanol. The Zn foil was then immersed in 0.2 M $SnCl_2$ solution ($SnCl_2$ in ethanol) for 5 min at 60 °C to form Sn layers via a chemical displacement reaction. The obtained Sn-coated Zn anodes were washed with ethanol and dried in a vacuum oven at 60 °C.

Preparation of gel electrolytes

PAM/$CuSO_4$ and PAM/$ZnSO_4$ gel: 3 g of acrylamide, 15 mg of $(NH_4)_2S_2O_8$, and 2 mg of N,N-Methylenebis (acrylamide) were added into 20 mL DI water and stirred at room temperature for 30 min. The solution was transferred into a mold and heated at 70 °C in an oven for 30 min to obtain the PAM gel. Finally, the flexible PAM gel was soaked in 1 M $CuSO_4$ (pH =1) and 2 M $ZnSO_4$ solution for 12 h to obtain PAM/$CuSO_4$ and PAM/$ZnSO_4$ gels respectively.

PVA/KOH gel: An aqueous solution of 20 wt% PVA was stirred at 90 °C for 1 h. The solution was poured into a mold and frozen at -30 °C for 12 h. The flexible PVA gel was then soaked in a solution of 6 M KOH + 0.3 M $Zn(Ac)_2$ for 12 h to obtain PVA/KOH gel. The thickness of the obtained gel electrolytes is around 2~3 mm.

Assembly of batteries and electrochemical characterization

Cu//Cu symmetrical and Cu//X (X=titanium foil (TF), Carbon cloth (CC), or Carbon paper (CP)) asymmetrical cells were tested using coin cells (CR2016-type). Commercial Cu foil was cut into circular sheets of 10 mm diameter to be directly used as Cu electrodes. $CuSO_4$ solutions and glass fiber (GFA) were used as the electrolyte and separator, respectively. Zn-Cu H-type cells were assembled in a purpose-made glass mold. The AEM Zn-Cu cell was assembled using 1x1 cm^2 Cu sheet, 1x1 cm^2 Sn-coated Zn sheet, 1 M $CuSO_4$ solution (pH =1), 2 M $ZnSO_4$ solution, and AEM (fumasep FAA-3-PK-130) as the cathode, anode, catholyte, anolyte, and separator respectively. The cathode-free and

anode-free cells were made by replacing the cathode or anode with CP. The alkaline-acid hybrid Zn-Cu cell was fabricated by replacing the anolyte and separator with 6 M KOH + 0.3 M $Zn(Ac)_2$ solution and BM (table S2). All battery testing was carried out using a Battery Testing System (CT-ZWJ-40S-T-1U) from Neware Technology Co., Ltd., Shenzhen, China.

Assembly of flexible Zn-Cu pouch cells

The commercial Cu foil (thickness: 100 μm) and the obtained Sn-coated Zn foil (thickness: 100 μm) were cut into the appropriate shape. Cu metal, PAM/$CuSO_4$, AEM, PAM/$ZnSO_4$, and Sn-coated Zn sheet were then stacked from bottom to top and sealed with aluminum plastic film to obtain the AEM Zn-Cu pouch cell. Similarly, Cu metal, PAM/$CuSO_4$, BM, PVA/KOH, and Sn-coated Zn sheet were stacked layer-by-layer to assemble the alkaline-acid hybrid Zn-Cu pouch cell. Regarding the large format pouch cells, a limited amount of Cu with a thickness of 15 μm was utilized to verify its practical feasibility.

Material characterization

SEM images and EDS elemental mapping were collected using a FE-SEM (JEOL-7100F) microscope at an acceleration voltage of 15 kV. The chemical states of the samples were investigated by XPS (Thermo Fisher Scientific- ESCALAB 250Xi). X-ray diffraction (XRD) patterns were collected on a D2 Advance X-ray diffractometer (Bruker) using a Cu Kα X-ray source. Fourier transform infrared (FT-IR) spectra were measured by using a Nicolet iS50 FTIR spectrometer. For the in-situ XRD measurements, we designed a cathode-free mould cell with a window to detect the Cu plating and stripping. The mould cells were constructed utilizing carbon cloth as the working electrode, 1 M $CuSO_4$ solution (with its pH either maintained or adjusted to 1 using H_2SO_4) as the electrolyte, Cu foil as the counter electrode, and cling film serving as the window. The in-situ cells were cycled at 2 mA cm^{-2}, 2 mAh cm^{-2}, within a 2θ range from 24° to 53°. During the testing process, signals were recorded every 110 seconds in a still mode.

Binding energy calculation

The theoretical simulations are based on first-principles calculations using density functional theory as implemented in the CASTEP software[1]. The electron exchange and correlation potentials are described by the generalized gradient approximation (GGA) with the Perdew, Burke, and Ernzerhof (PBE) functional. The kinetic energy cutoff of 400 eV is used for the plane-wave basis sets. The surface models are constructed by a 4×4 five-layer Sn (020) and a Zn (011) supercell respectively. The bottom

two layers are fixed and only the top three layers are freely relaxed. The initial position of an absorbed Zn atom is placed at the top, bridge and tetragonal sites in order to find out the most stable structure. A 3×3×1 k-point grid with the Monkhorst-Pack scheme is used for the Brillouin zone sampling, and the convergence tolerance is reached when the Hellman-Feynman forces on an individual atom are less than 0.03 eV/Å. The binding energy (E_b) of a Zn atom on the surface is derived by the following formula:

$$E_b = E_{total} - E_{surf} - E_{Zn}$$

where E_{total}, E_{surf}, and E_{Zn} are the total energies of the optimised surface with adsorbed atom, clean surface, and single Zn atom, respectively.

In order to demonstrate the stability of Cu deposition on CP, we calculated the binding energy of Cu interacting with CP (graphite) and Ti substrates, respectively. The calculation involves supercells of the 7 layers of Cu (111), and substrates with 4 layers of graphite or 7 layers of Ti (001). The supercell lattice constants are set to those of substrate lattices, while the vertical metal distances are allowed to relax. A roughly 20 Å vacuum spacing is retained between slabs. The interface binding energy is calculated as

$$E_{binding} = (E_{Cu/substrate} - n \times E_{Cu} - m \times E_{substrate})/\#substrate$$

where $E_{Cu/substrate}$, $E_{Cu}(1 \times 1)$ and $E_{substrate}(1 \times 1)$ are the total energy of the contact supercell, the Cu(1×1) surface slab and substrate(1×1) slab, respectively. #substrate is the number of substrate formula units.

2. Supplementary Figures and Tables

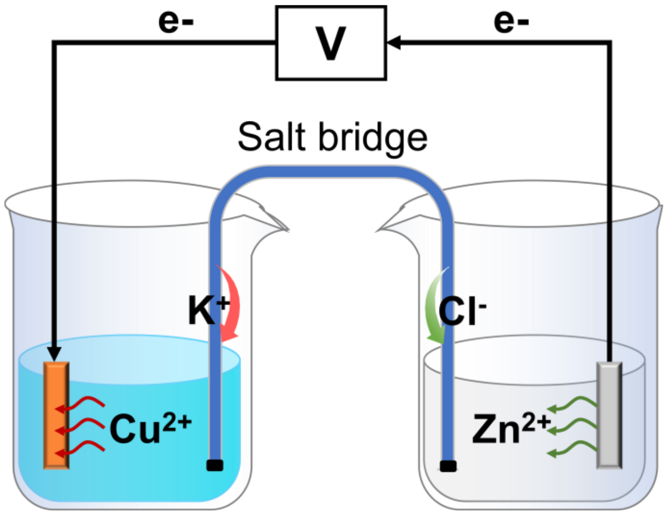

Fig. S1. Schematic illustration of a typical Daniel cell.

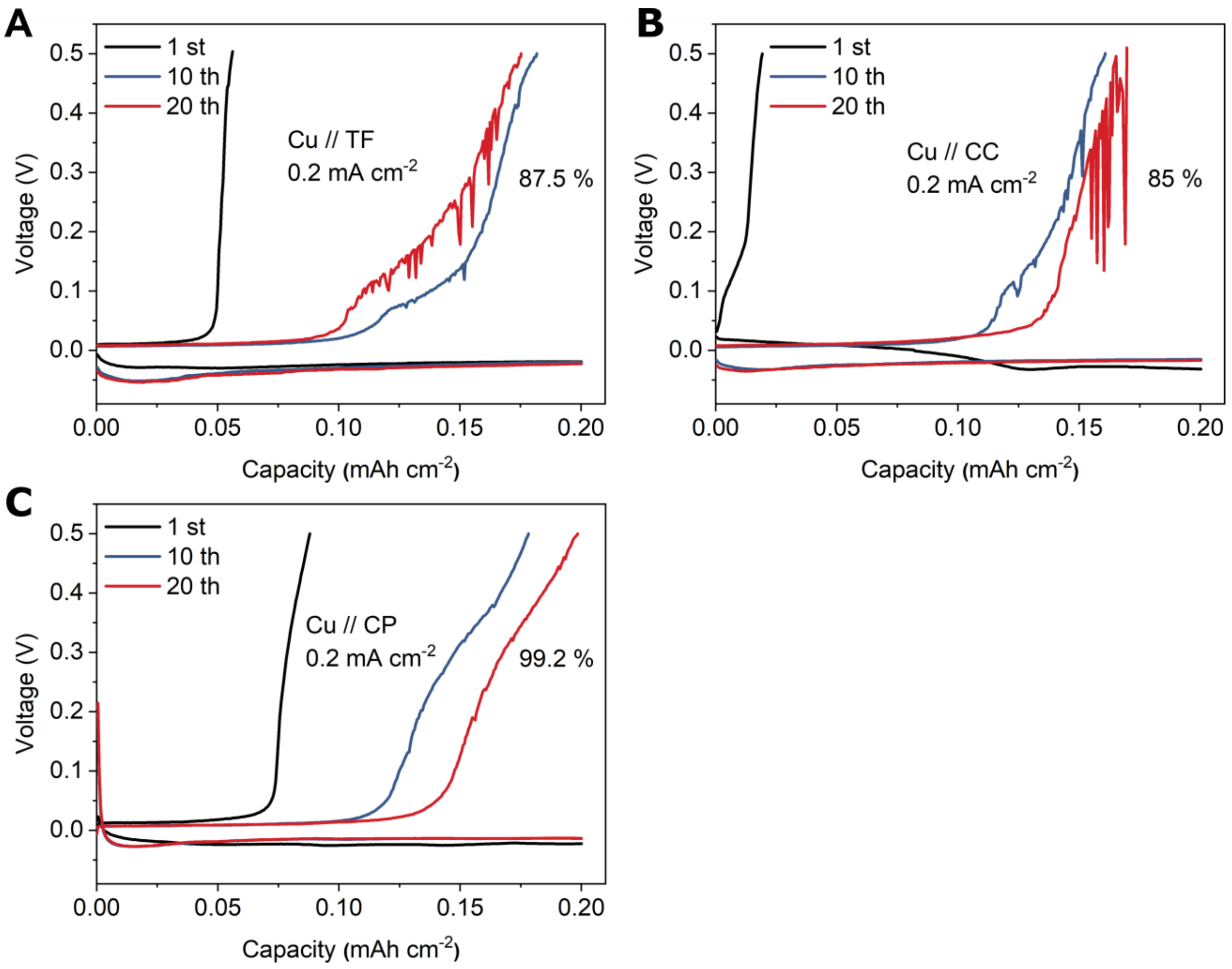

Fig. S2. Galvanostatic voltage profiles of (A) Cu//Titanium foil (TF), (B) Cu//Carbon cloth (CC), and (C) Cu//Carbon paper (CP) asymmetrical cells at 0.2 mA cm^{-2} with a plating capacity of 0.2 mAh cm^{-2}. The Coulombic efficiencies of Cu plating/stripping were investigated by asymmetric cells using Cu metal as the counter electrode and TF, CC, or CP as the working electrodes, in which CP exhibits the highest Coulombic efficiency and stability.

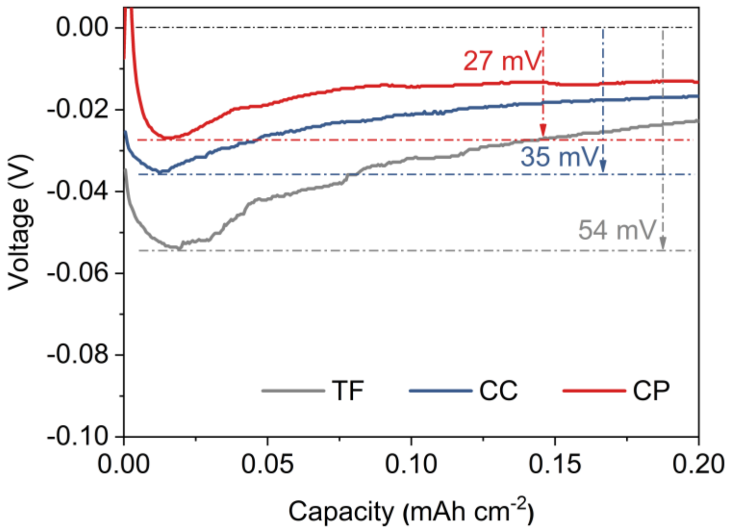

Fig. S3. Nucleation overpotentials of Cu//TF, Cu//CC, and Cu//CP asymmetrical cells. The electrodeposition of Cu on CP shows the lowest nucleation overpotential (27 mV) compared to CC (35 mV), and TF (54 mV), indicating the lower deposition barrier when utilizing the CP substrate.

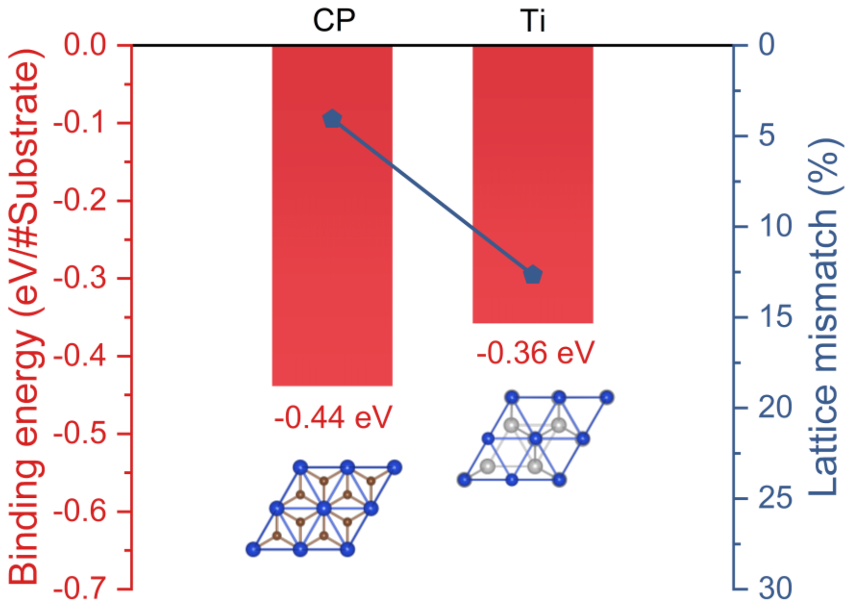

Fig. S4. The mismatch and binding energy between Cu and different substrates. The DFT calculation results show that the lattice mismatch of CP/Cu is 4.06%, which is much lower than that of Ti/Cu (12.66%); the binding energy of CP/Cu is -0.44 eV per CP formula unit, which is lower than Ti/Cu of -0.36 eV per Ti formula unit. Therefore, owing to the low lattice matching and binding energy between Cu and CP substrate, Cu exhibits good stability when electrodeposited on the CP substrate.

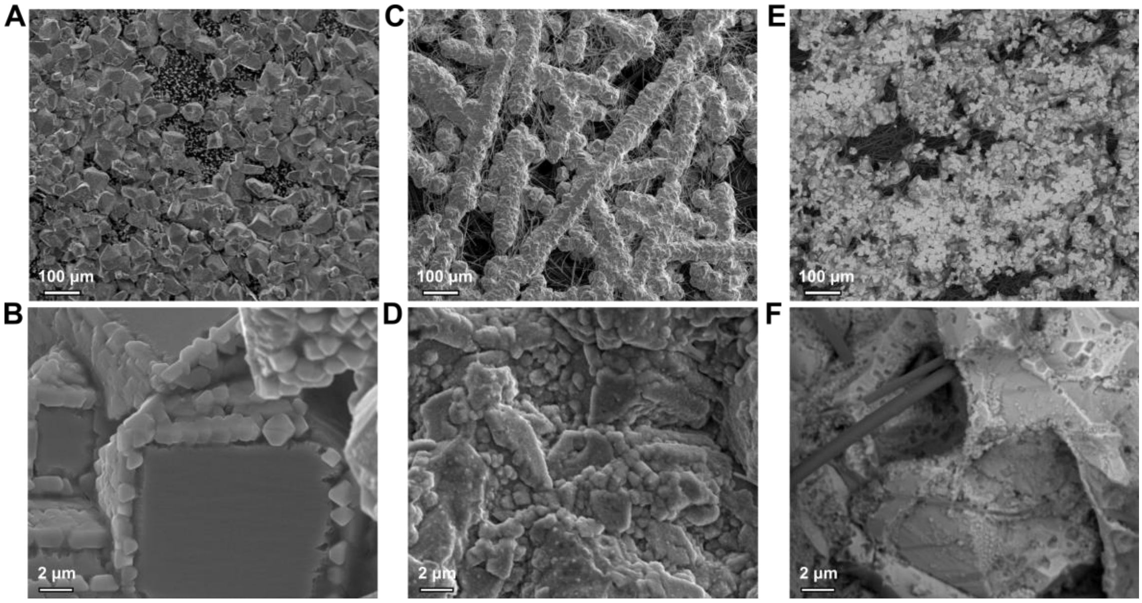

Fig. S5. SEM images of electrodeposited Cu on (A-B) CP, (C-D) CC, and (E-F) TF. The plated Cu on both CP and CC displays a more regular shape than that on TF. Furthermore, the Cu deposited on the CP substrate showcases a more uniform and compact morphology compared to that on CC. These findings underscore the advantage of using CP as the preferred substrate for Cu plating.

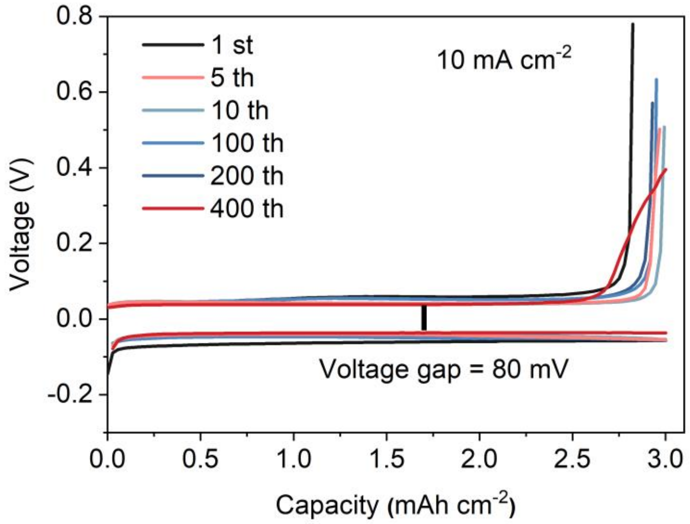

Fig. S6. Galvanostatic voltage profiles of Cu//CP asymmetrical cell in 1 M $CuSO_4$ electrolyte (pH =1) at 10 mA cm^{-2}, 3 mAh cm^{-2}. The typical plateaus for Cu plating/stripping show a high Coulombic efficiency (~99.7 %) and low overpotential (~80 mV) for 400 cycles.

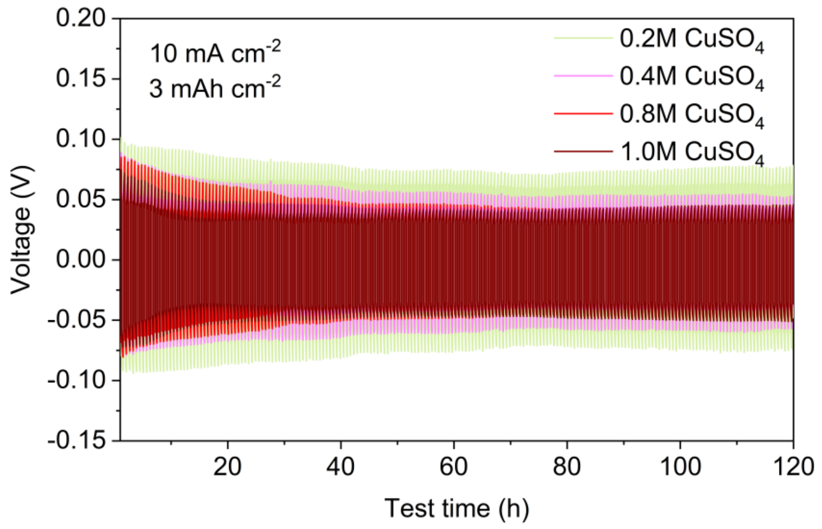

Fig. S7. The time-voltage curves for Cu//Cu symmetrical cells in different electrolyte concentrations at 10 mA cm^{-2} with a plating capacity of 3 mAh cm^{-2}. The overpotentials are about 160, 120, 100, and 80 mV at 0.2 M, 0.4 M, 0.8 M, and 1 M $CuSO_4$ electrolyte (pH =1), respectively.

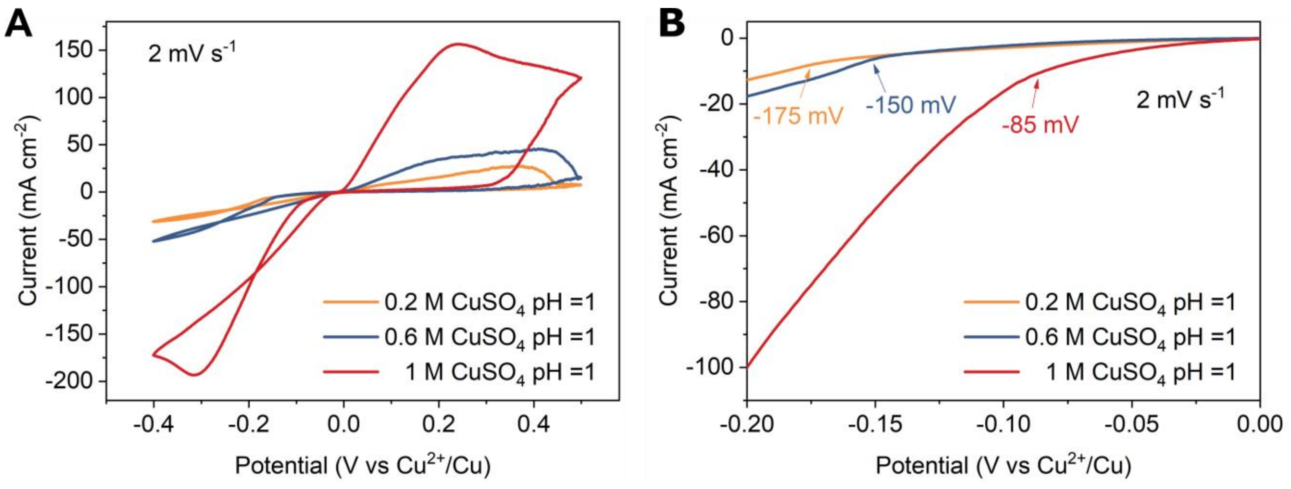

Fig. S8. (A) Cyclic voltammograms (CV) curves of Cu/CP asymmetrical cells using electrolytes of different concentrations at scan rate of 2 mV s^{-1}. (B) Zoomed-in view of (A). The higher concentration electrolyte exhibits a significantly higher response current during the CV test, indicating better Cu plating/stripping kinetics. Additionally, the 1 M $CuSO_4$ (pH =1) electrolyte demonstrates earlier Cu reduction compared to the lower concentration electrolytes. These results are consistent with the lower overpotential observed in Cu//Cu symmetrical cells (fig. S4).

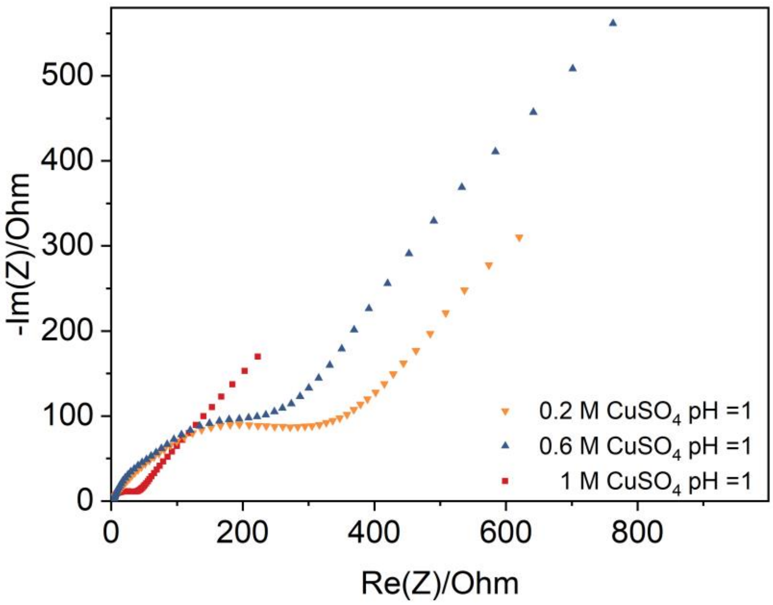

Fig. S9. Nyquist plots of electrochemical impedance spectroscopy (EIS) for Cu/CP asymmetrical cells using electrolytes of different concentrations. The 1 M $CuSO_4$ (pH =1) electrolyte exhibits much lower interfacial impedance compared to the lower concentration electrolytes, indicating faster charge transfer.

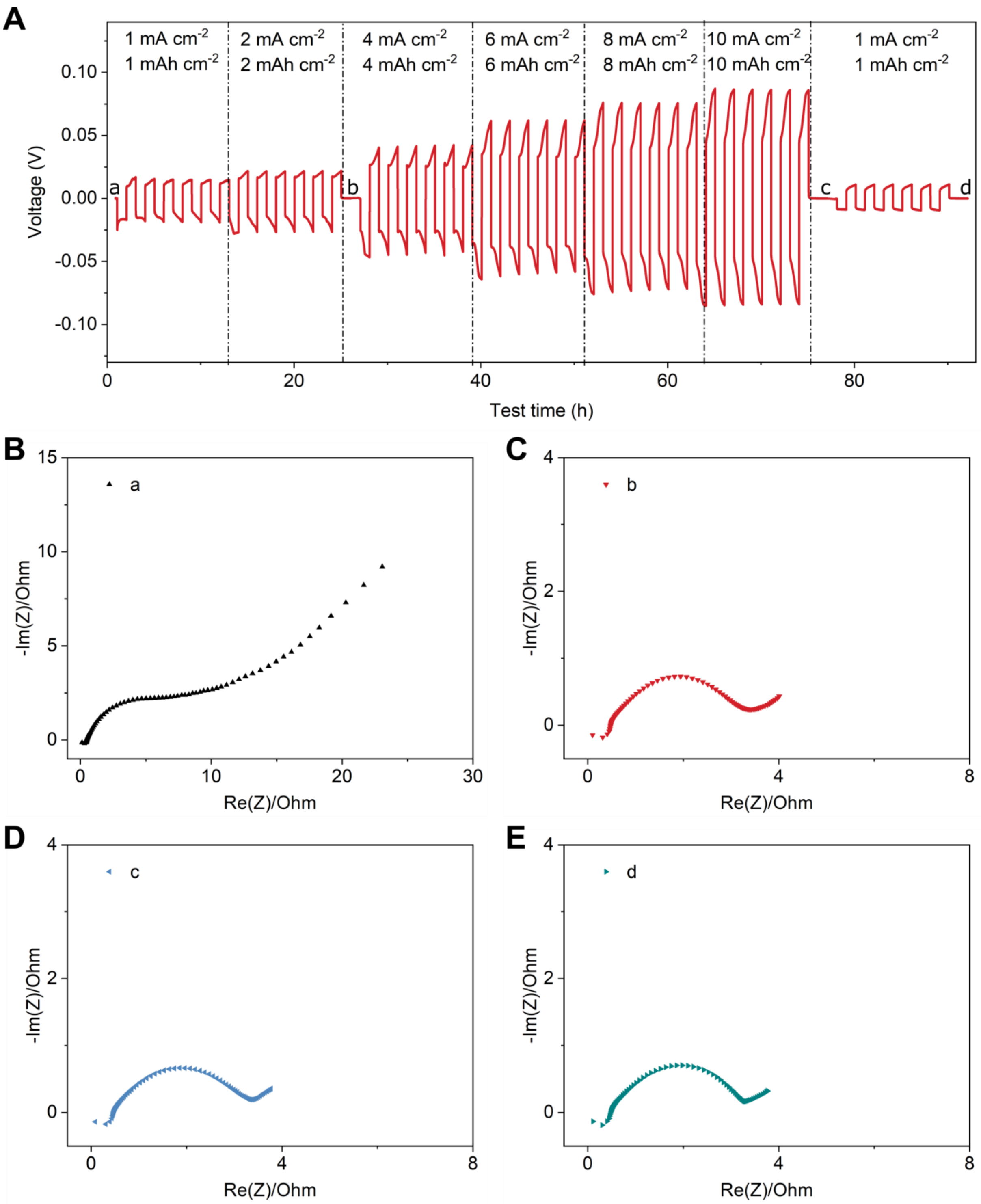

Fig. S10. (A) Time-voltage curve of the Cu//Cu symmetrical cell under various current densities, along with the Nyquist plots from EIS measurements at (B) point a, (C) point b, (D) point c, and (E) point d. The Cu/Cu symmetrical cell initially displays an R_{ct} value of approximately 10 Ω. This value reduces to approximately 3 Ω after cycling at low current densities and remains consistent throughout the subsequent rate test, leading to a reduced overpotential as evident in the concluding voltage curve of the rate test. This observation confirms that no soft short circuit occurred during the rate test[2].

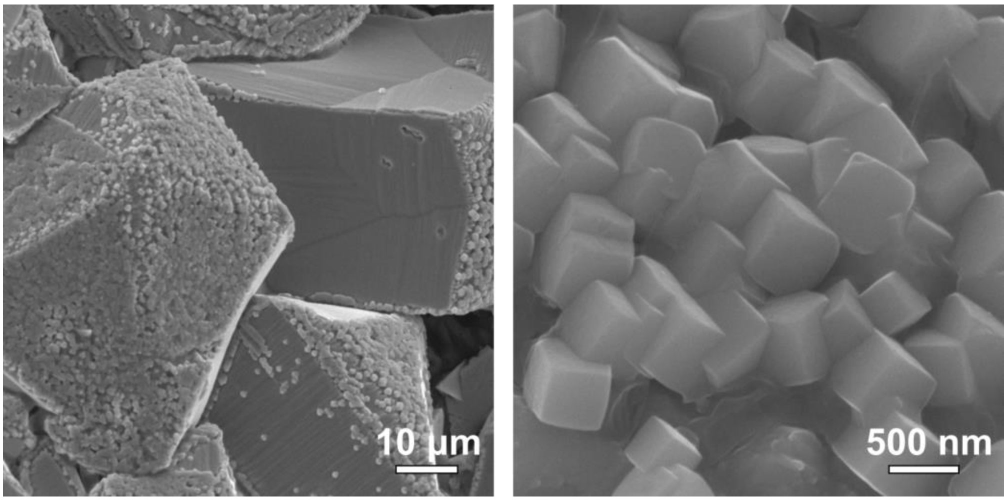

Fig. S11. SEM images of Cu surface after 400 cycles at different magnifications. The deposited Cu shows a uniform cubic stacking morphology without dendrite growing.

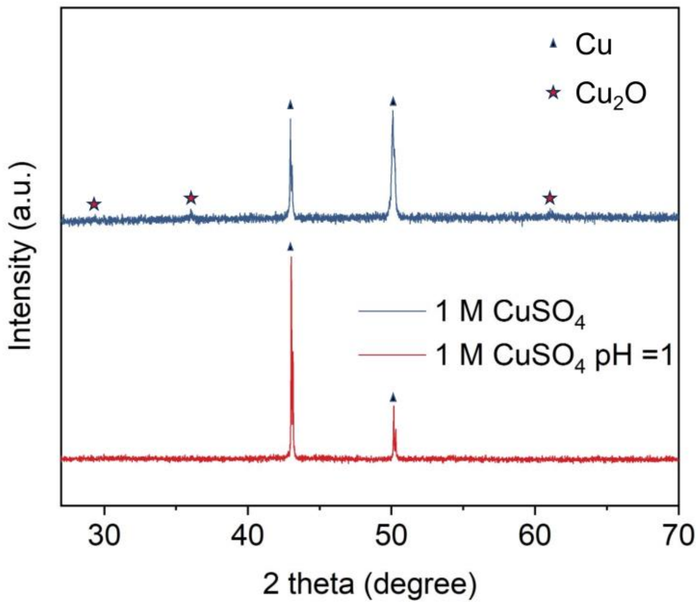

Fig. S12. XRD patterns of Cu foil after 20 cycles in different electrolytes. The Cu foil cycled in 1 M $CuSO_4$ displays additional peaks corresponding to Cu_2O, suggesting the formation of CuO_x during cycling. Conversely, when cycled in 1 M $CuSO_4$ with adjusted pH of 1, the Cu foil shows no such additional peaks. It is worth noting that the ex-situ XRD tests were conducted on washed Cu foils from disassembled cells, the peak intensity of CuO_x is lower than that observed in in-situ XRD tests.

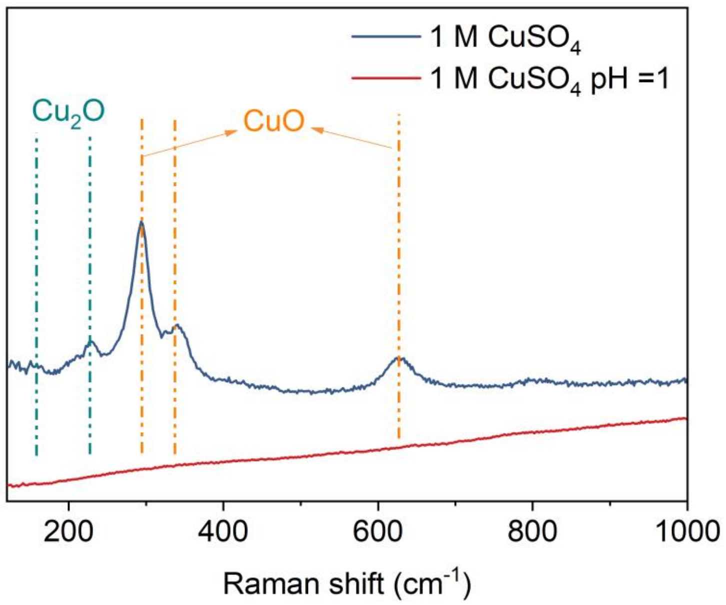

Fig. S13. Raman spectra of Cu foil after 20 cycles in different electrolytes. The Cu foil cycled in 1 M $CuSO_4$ shows additional peaks of Cu_2O (150 cm^{-1} and 220 cm^{-1})[3] and CuO (293 cm^{-1}, 342 cm^{-1}, and 623 cm^{-1})[4], while the Cu foil cycled in 1 M $CuSO_4$ with adjusted pH of 1 exhibits no additional peaks.

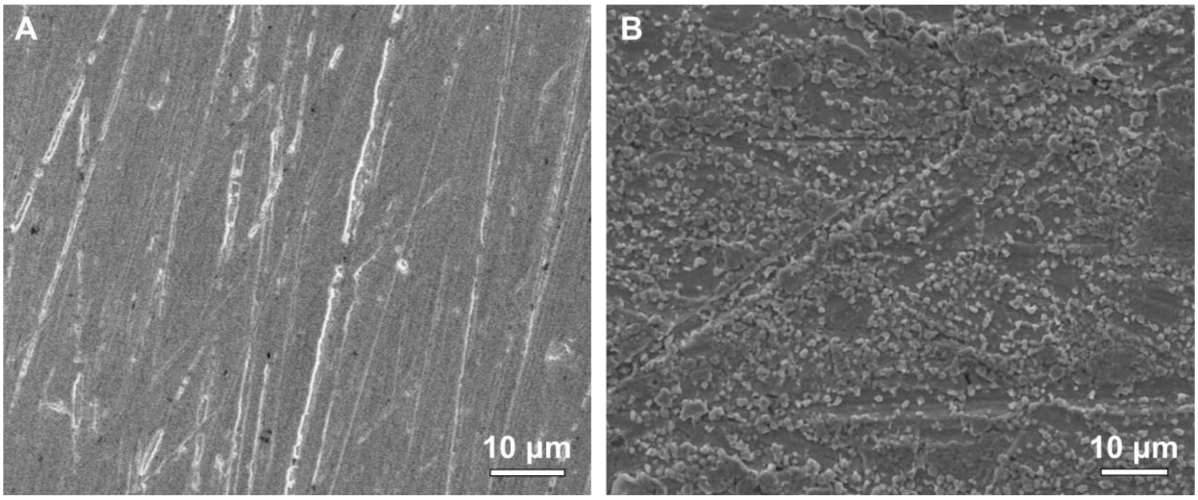

Fig. S14. SEM images of **(A)** bare Zn and **(B)** Sn-coated Zn. After modification, the Zn foil was covered by the dense Sn layer composed of irregular Sn particles.

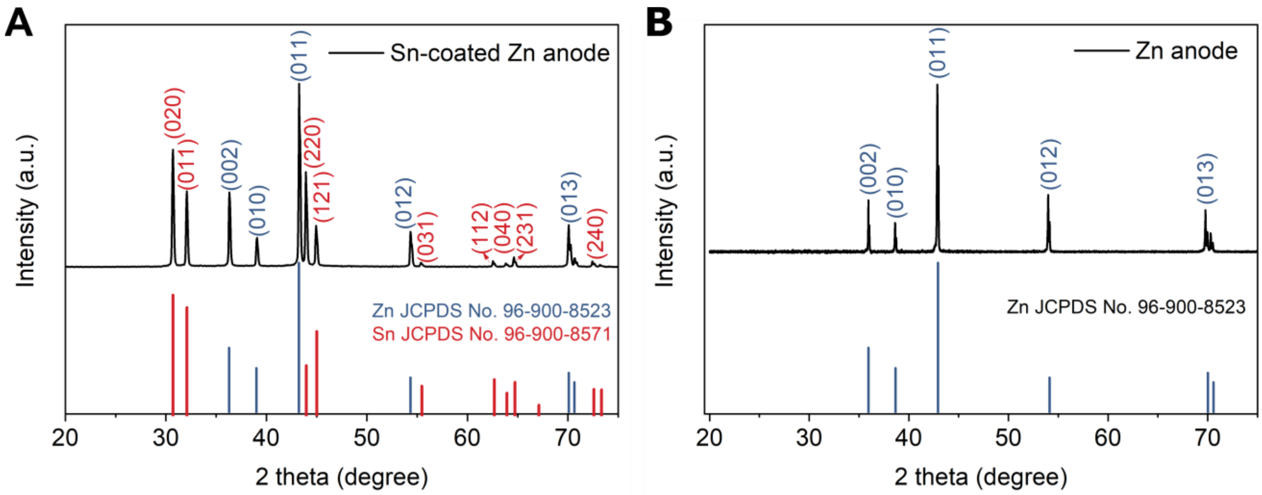

Fig. S15. XRD patterns of (A) bare Zn and (B) Sn-coated Zn. The Sn-coated Zn anode shows additional peaks at 30.7°, 32.1°, 43.9°, 45°, 55.5°, 62.7°, 63.9°, 64.7°, 72.6°, corresponding to the (020), (011), (220), (121), (031), (112), (040), (231), (240) facets of Sn (JCPDS No. 96-900-8571).

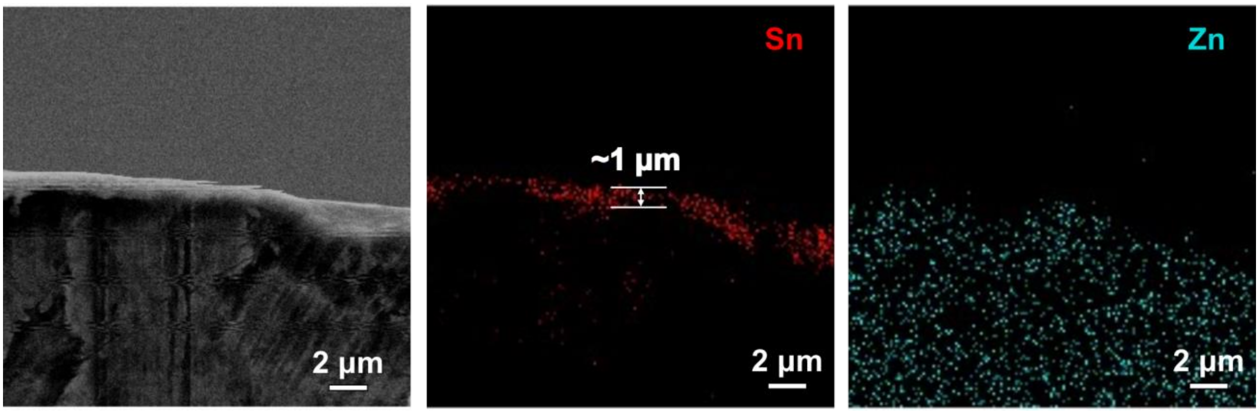

Fig. S16. Cross-sectional SEM image of Sn-coated Zn anode and corresponding EDS mapping images. The thickness of the Sn layer on the Zn foil is ~1 μm.

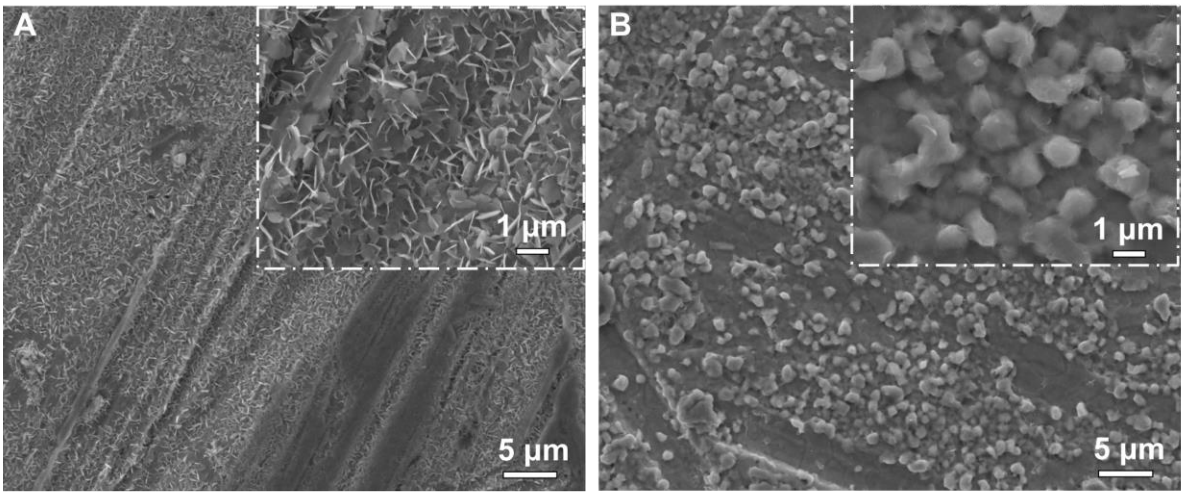

Fig. S17. SEM images of (A) bare Zn and (B) Sn-coated Zn anodes after immersion in 2 M $ZnSO_4$ electrolyte for 1 day. The bare Zn was covered by numerous flakes after immersion, which is the typical by-product of Zn corrosion in a mild acidic electrolyte[5]. In comparison, the Sn-coated Zn anode shows an unchanged morphology after immersion, indicating the suppressed Zn corrosion reaction.

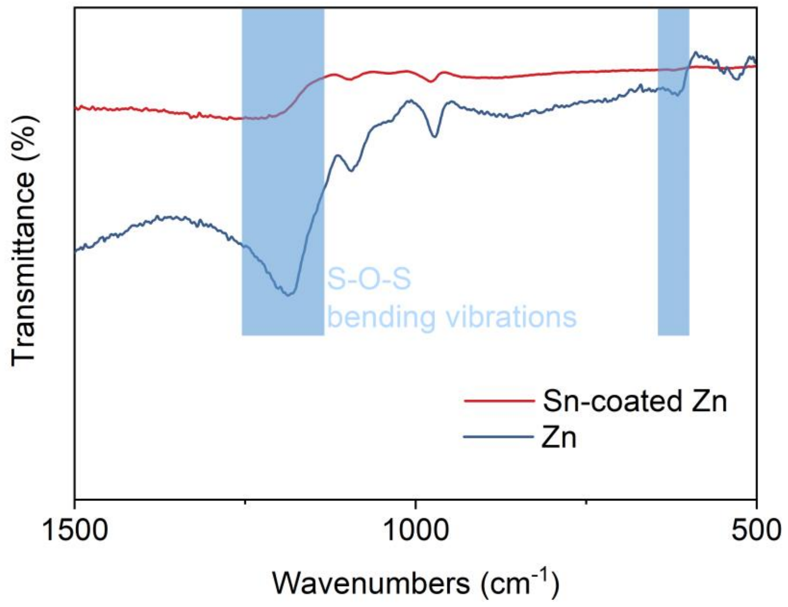

Fig. S18. FT-IR spectra of bare Zn (red) and Sn-coated Zn (green) anodes after immersion in 2 M $ZnSO_4$ solution for 1 day. The soaked Zn shows strong absorption peaks at ~1160 and ~600 cm^{-1}, which are attributed to the S-O stretching vibration and the O-S-O bending vibration of SO_4[2-6], corresponding to the formation of $Zn_4SO_4(OH)_6 \cdot xH_2O$[7]. The soaked Sn-coated Zn anode shows much weaker peaks compare to the bare Zn, which is consistent with the SEM results.

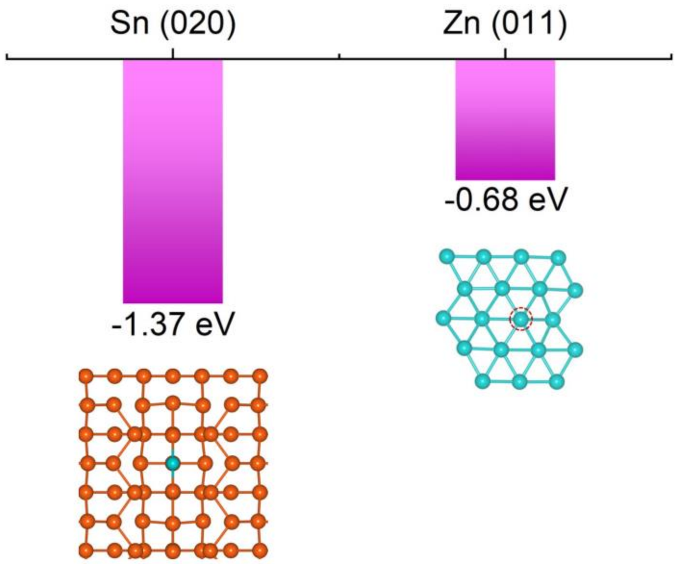

Fig. S19. The binding energies of a Zn atom on the surfaces of Sn (020) and Zn (011). The Sn (020) (-1.37 eV) shows a higher binding energy for Zn atoms than the Zn (011) (-0.68 eV), indicating that Sn has a good Zn affinity and Zn will preferentially deposit on Sn. The high Zn affinity of Sn can facilitate the deposition of Zn and drive heterogeneous nucleation of Zn, leading to uniform deposition of Zn[8, 9].

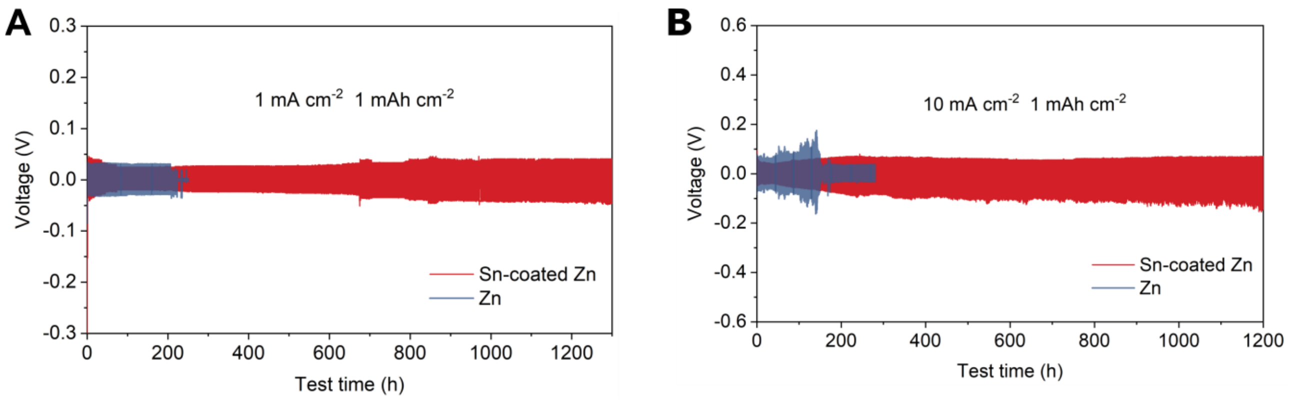

Fig. S20. Long-term time-voltage curves for bare Zn anode and Sn-coated Zn anode in symmetrical cells at (A) 1 mA cm^{-2}, 1 mAh cm^{-2} and (B) 10 mA cm^{-2}, 1 mAh cm^{-2}.

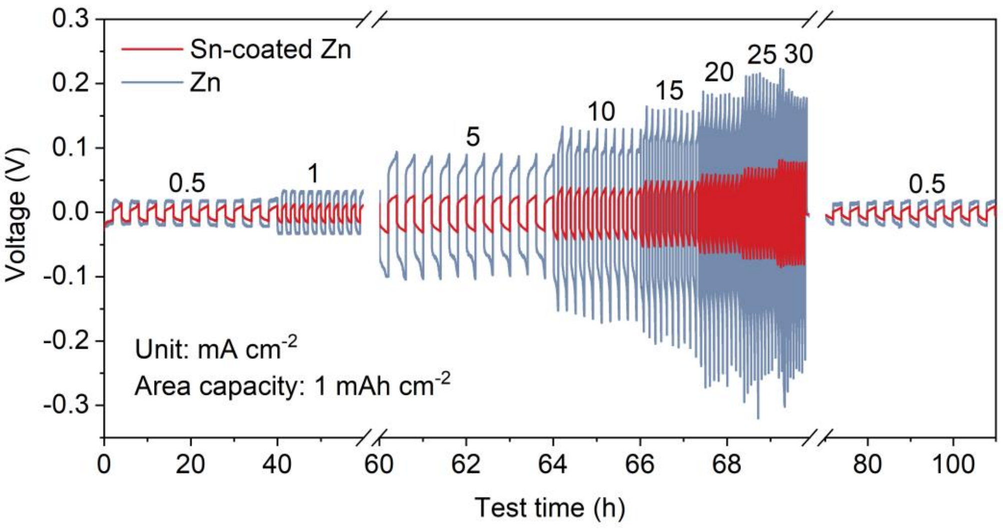

Fig. S21. Rate performance of bare Zn and Sn-coated Zn anodes in symmetrical cells at various current densities.

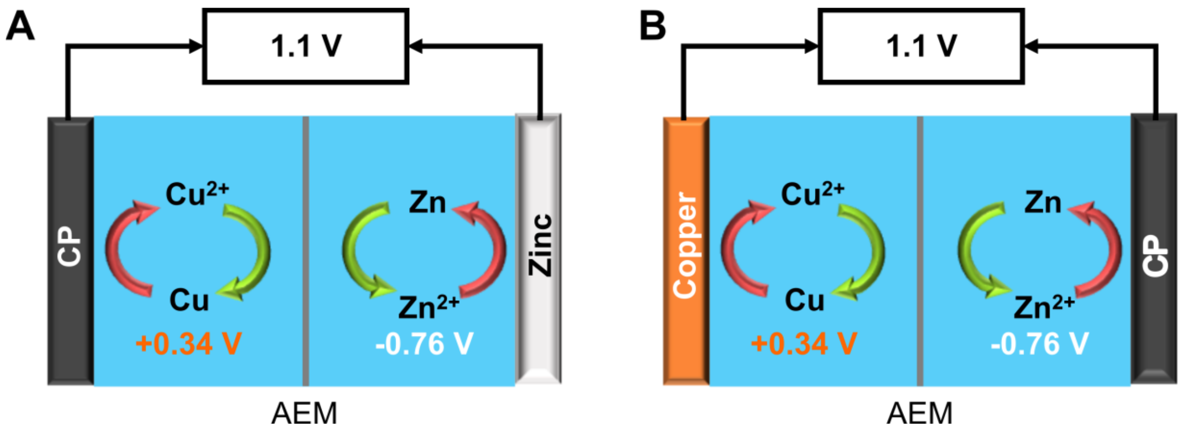

Fig. S22. Schematic diagrams for (A) the cathode-free and (B) anode-free Zn-Cu batteries.

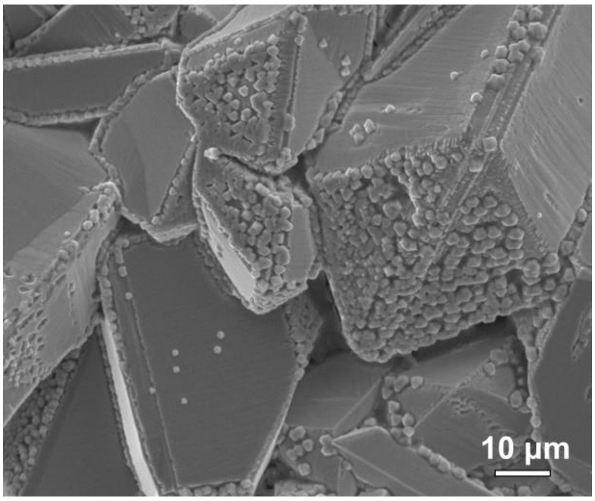

Fig. S23. SEM image of the CP of the cathode-free cell with 1 M $CuSO_4$ (pH =1) electrolyte after being discharged for 24 h. The deposited Cu exhibits a compact morphology formed by the regular stacking of small Cu cubes.

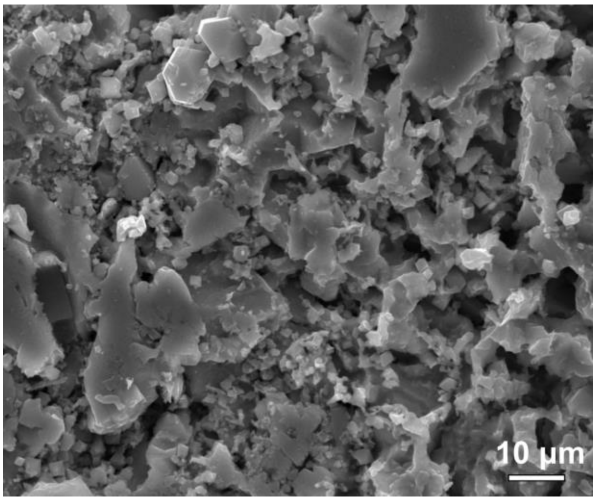

Fig. S24. SEM image of the CP of the cathode-free cell with neutral 1 M $CuSO_4$ electrolyte after being discharged for 24 h. The deposited Cu shows an uneven morphology consisting of irregular Cu.

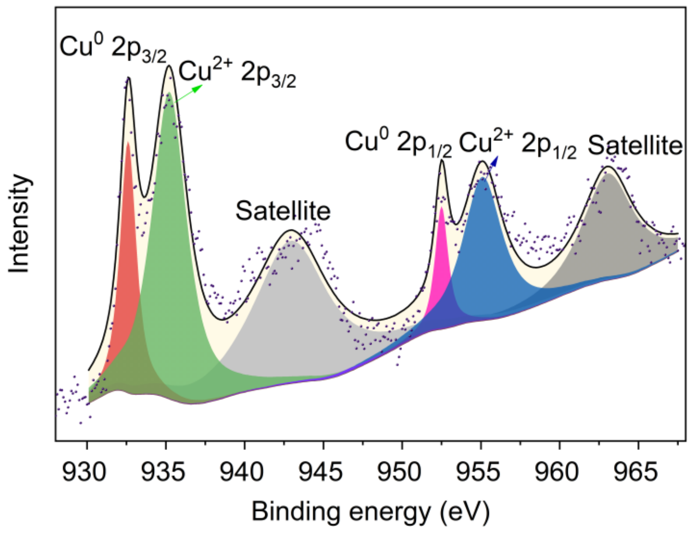

Fig. S25. XPS spectra of Cu 2p for CP after being discharged in neutral 1 M $CuSO_4$. The deposited Cu exhibits strong peaks at 934.7 eV and 954.3 eV, indicating that a significant amount of CuO was formed.

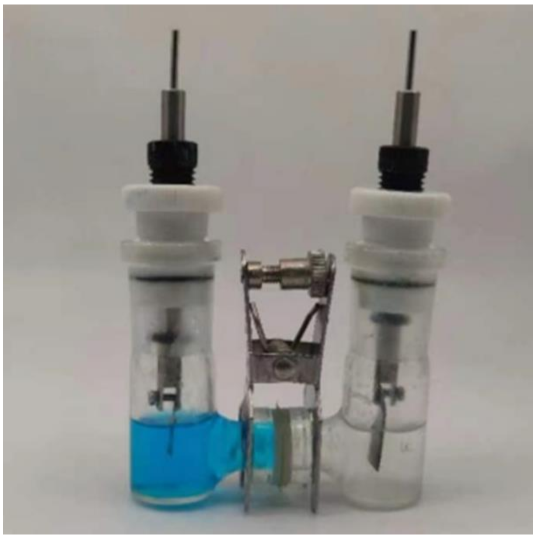

Fig. S26. The digital photo of the AEM Zn-Cu mould cell. The left tube contains the Cu metal electrode immersed in 1 M $CuSO_4$ (pH =1), the right tube holds the Sn-coated Zn metal electrode immersed in 2 M $ZnSO_4$, and the AEM is used as the separator.

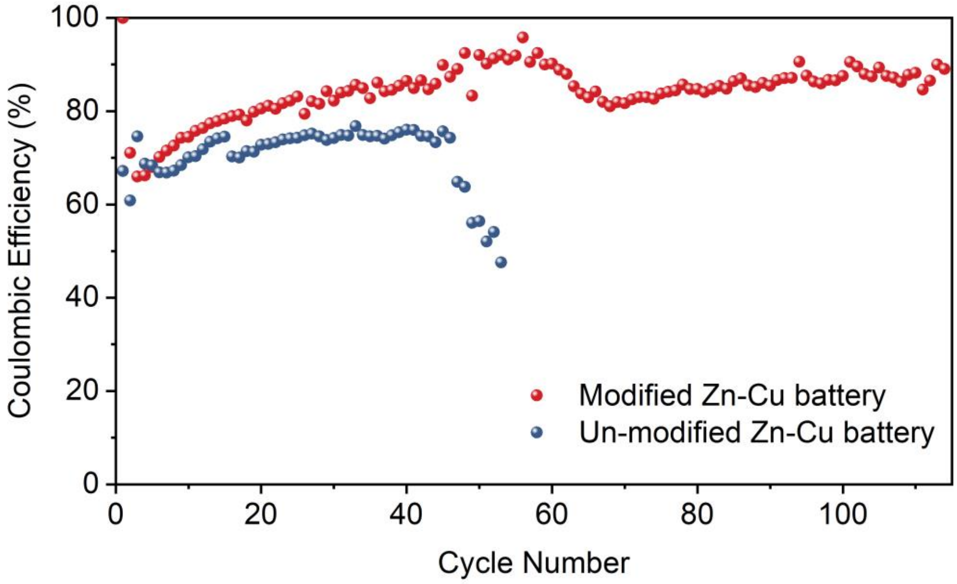

Fig. S27. The Coulombic efficiencies of modified AEM Zn-Cu battery (1 M $CuSO_4$ pH =1, Sn-coated Zn metal anode) and un-modified AEM Zn-Cu battery (1 M $CuSO_4$, bare Zn metal anode). The Coulombic efficiency of the modified cell reaches ~92% within 50 cycles and is stable for more than 100 cycles. In comparison, the un-modified cell can only survive for less than 50 cycles with low Coulombic efficiency of ~ 70%.

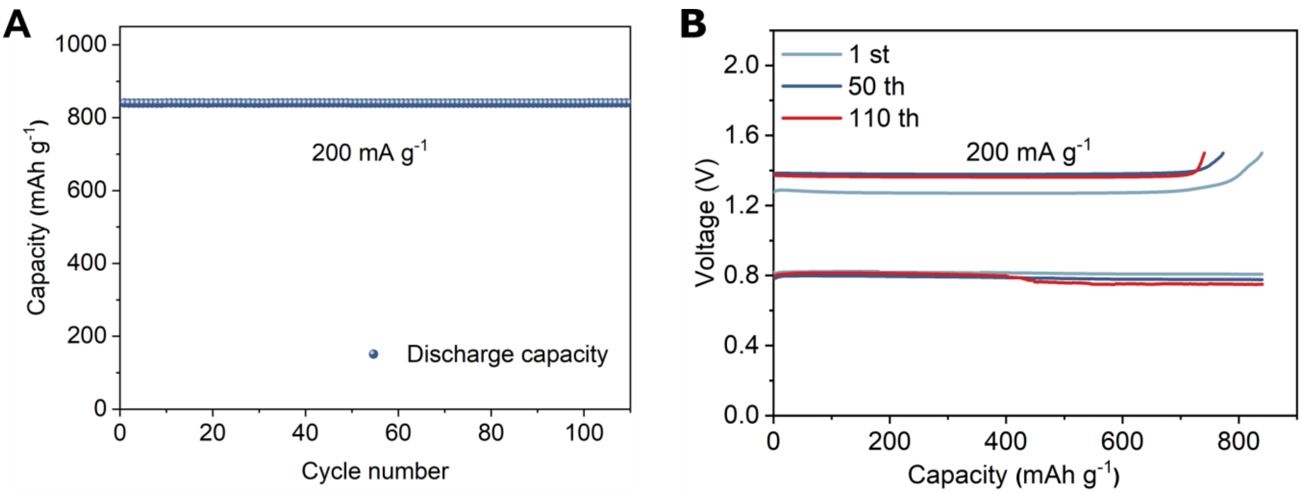

Fig. S28. Specific capacity of the Cu metal cathode. (A) Cycling performance at the current density of 200 mA g^{-1} and (B) the corresponding GCD curves.

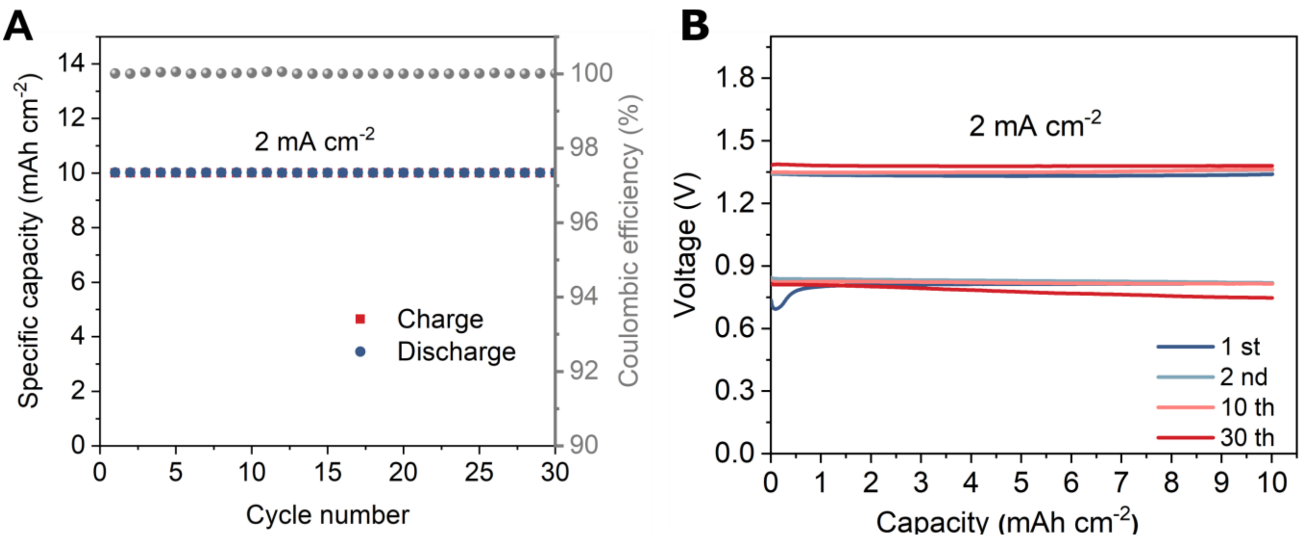

Fig. S29. (A) Cycling performance of the AEM Zn-Cu mold cell at 2 mA cm^{-2} with a high area capacity of 10 mAh cm^{-2} and (B) the corresponding GCD curves.

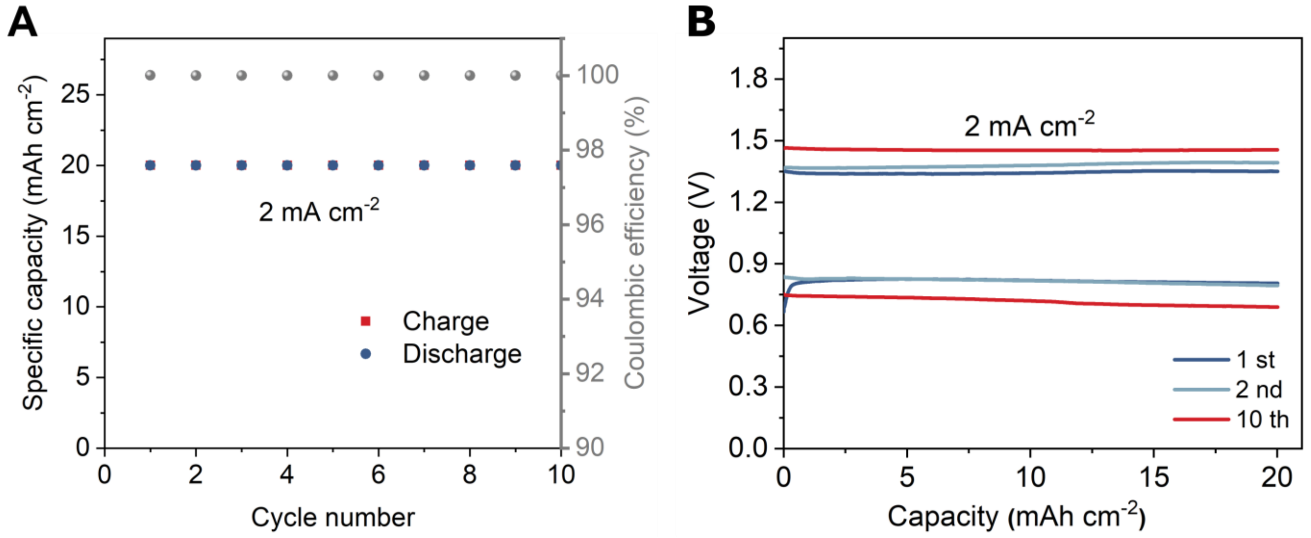

Fig. S30. (A) Cycling performance of the AEM Zn-Cu mold cell at 2 mA cm^{-2} with a high area capacity of 20 mAh cm^{-2} and (B) the corresponding GCD curves.

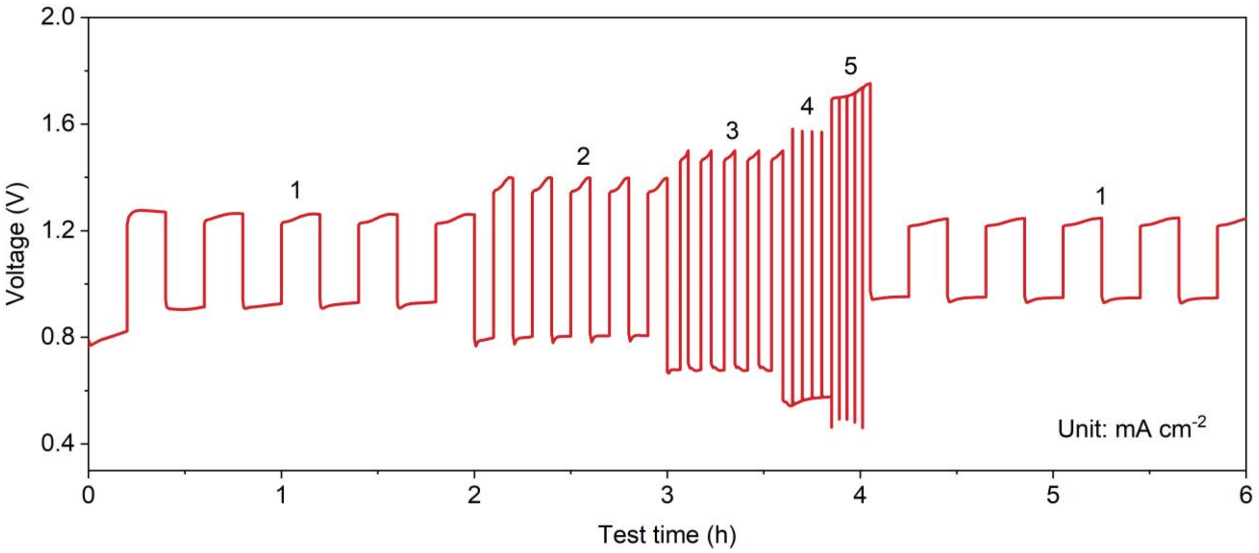

Fig. S31. Rate performance of the AEM Zn-Cu cell in the H-type mold cell.

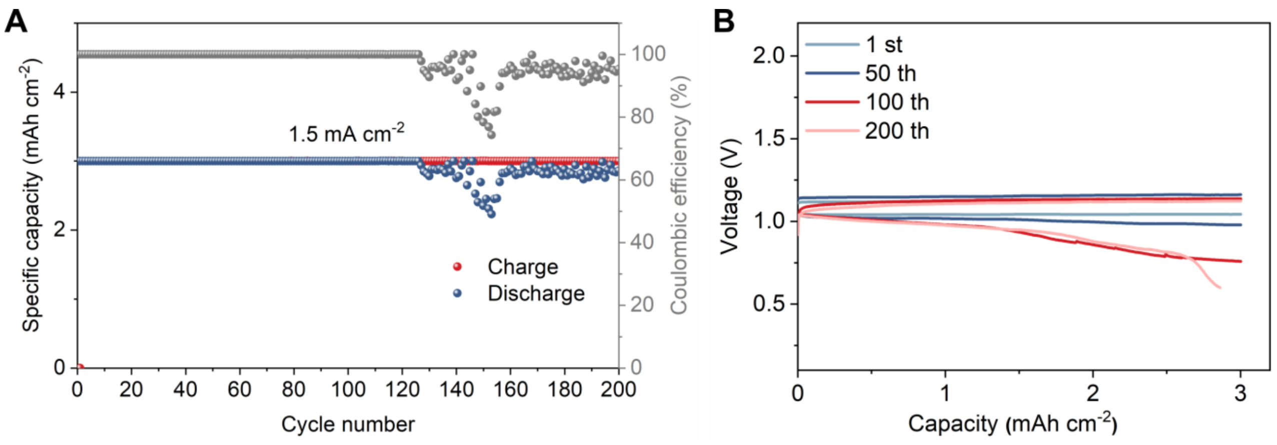

Fig. S32. (A) Cycling performance of the AEM Zn-Cu pouch cell at 1.5 mA cm^{-2} and (B) the corresponding GCD curves of 1st, 50th, 100th, and 200th cycles.

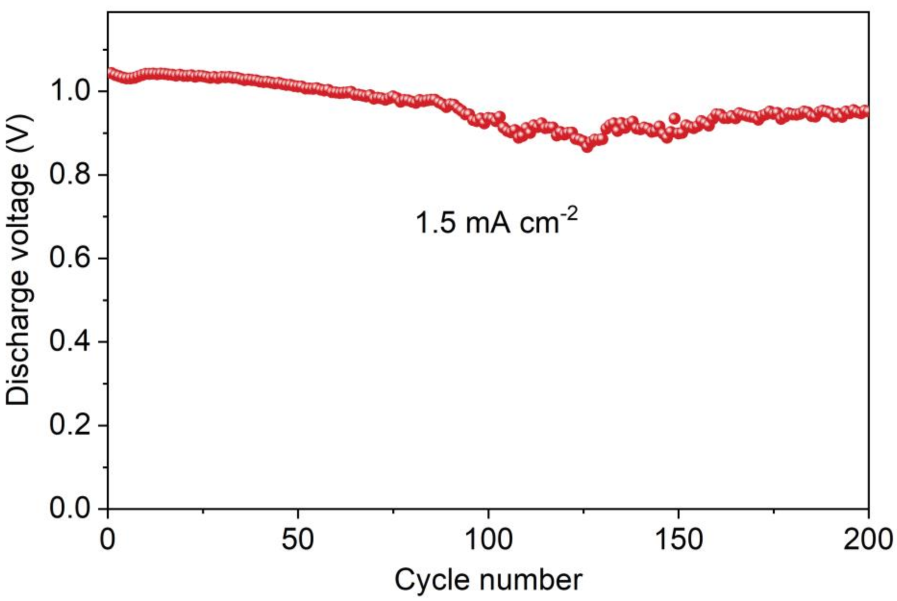

Fig. S33. The discharge voltage of the AEM Zn-Cu pouch cell at 1.5 mA cm^{-2}.

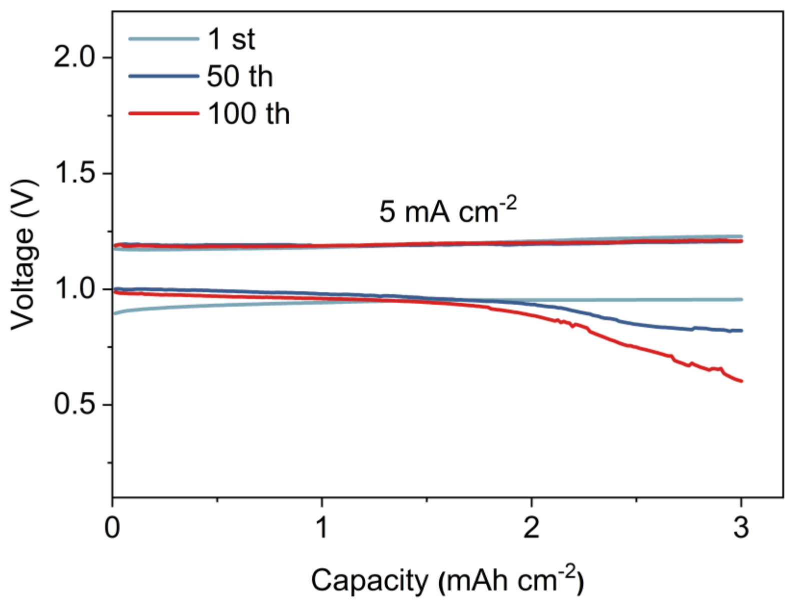

Fig. S34. GCD curves of the AEM Zn-Cu pouch cell at 5 mA cm^{-2} for 1st, 50th, and 100th cycles.

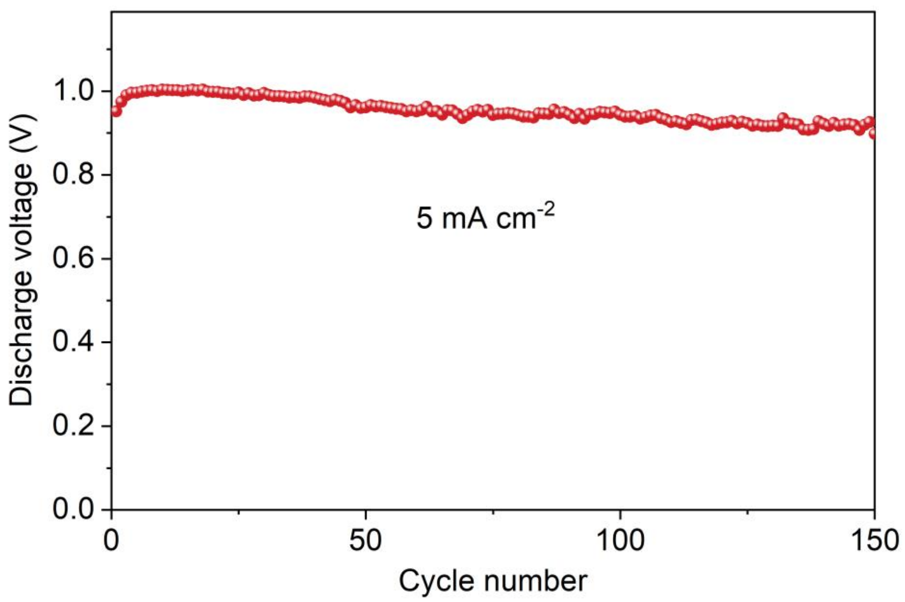

Fig. S35. The discharge voltage of the AEM Zn-Cu pouch cell at 5 mA cm^{-2}.

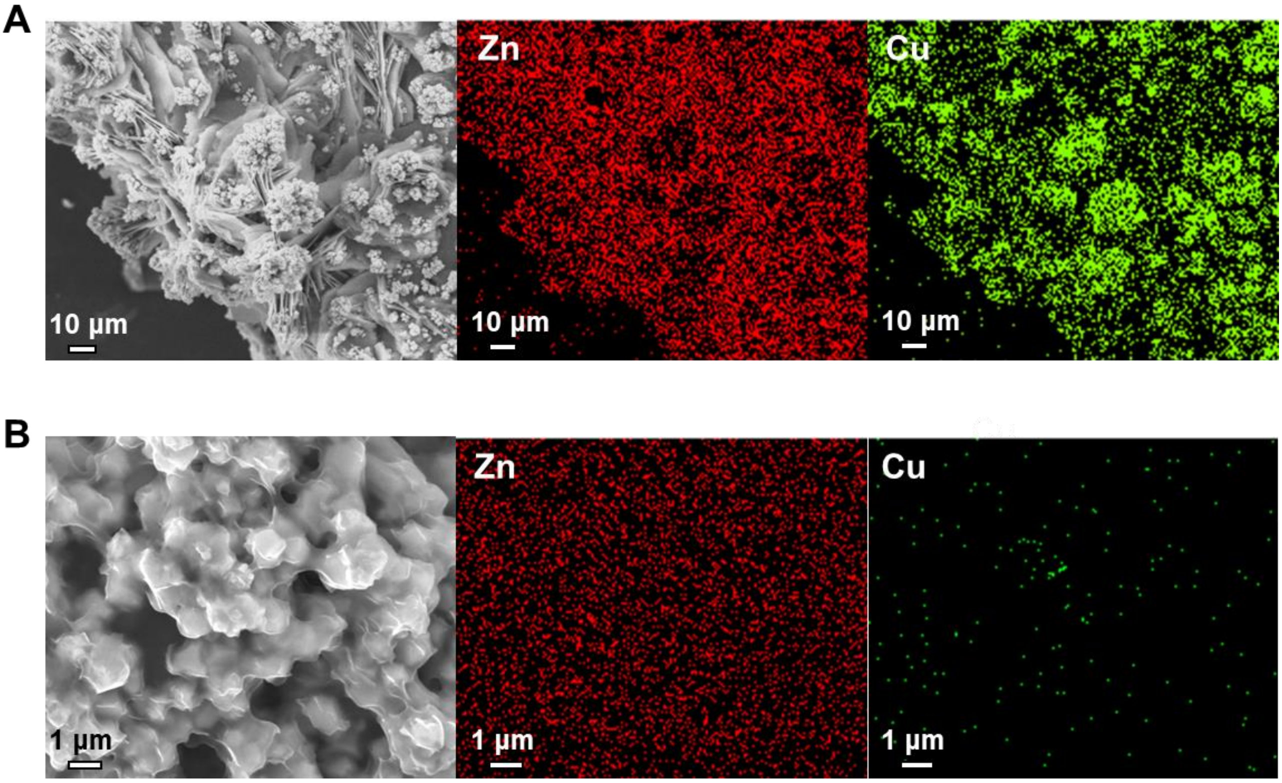

Fig. S36. (A) EDS elemental mappings of cycled Zn anode in the AEM Zn-Cu mold cell with liquid electrolytes and (B) cycled Sn-coated Zn anode in the AEM Zn-Cu pouch cell with gel electrolytes. The cycled Zn anode in the mold cell shows obvious Cu replacement due to the Cu^{2+} crossover, while the Sn-coated Zn anode cycled in gel electrolyte shows significantly suppressed Cu replacement. This demonstrates that the crossover effect can be reduced in our pouch cell design.

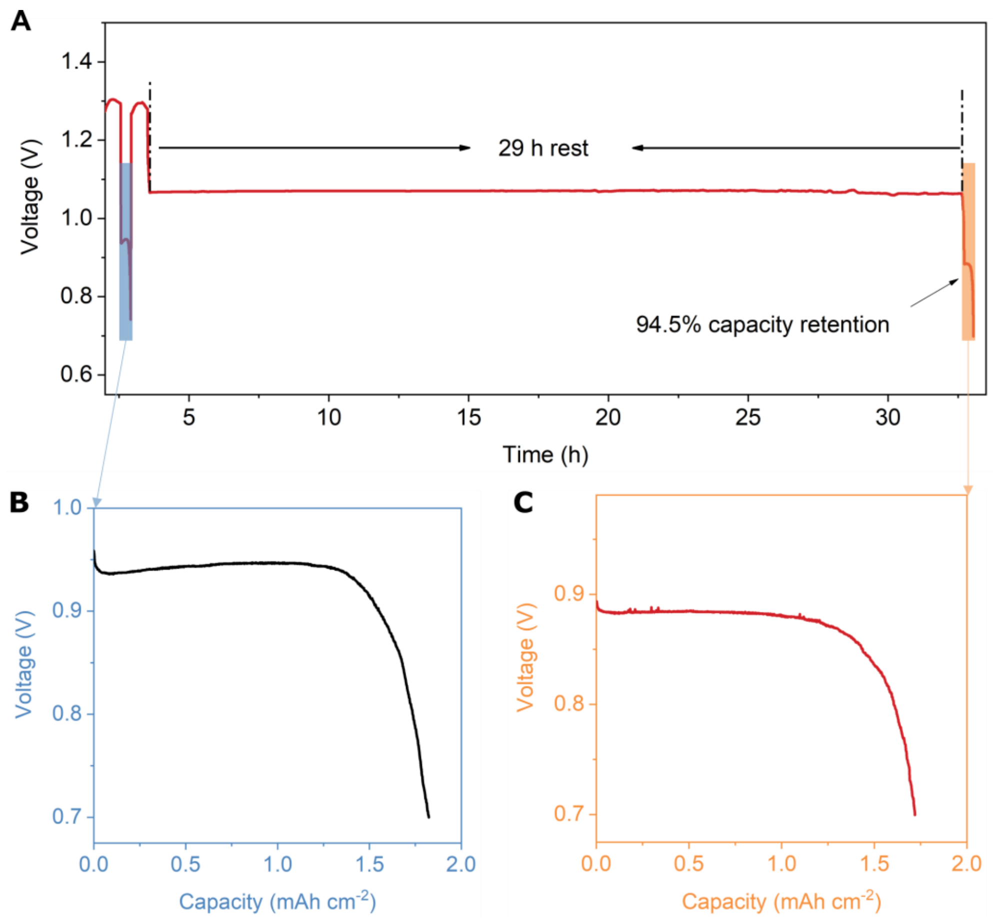

Fig. S37. Self-discharge test of the anode-free AEM Zn-Cu battery. The anode free AEM Zn-Cu pouch cell was assembled by replacing the Zn metal anode with a Sn-coated Ti foil to evaluate the self-discharge of the AEM Zn-Cu cell. The cell exhibited a specific capacity of 1.72 mAh cm^{-2} after 29 hours of rest, which corresponds to a 94.5% capacity retention compared to the discharge capacity of 1.82 mAh cm^{-2} before the rest period.

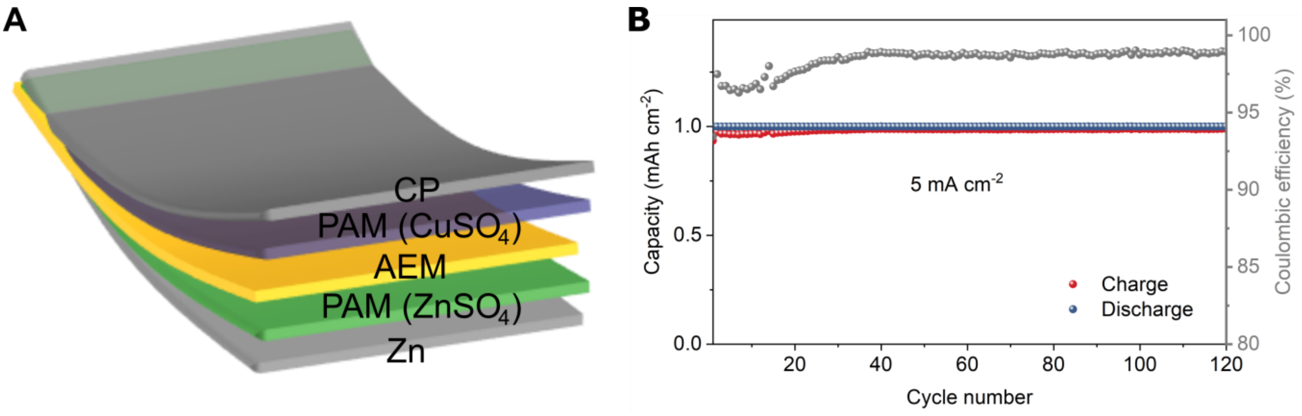

Fig. S38. (A) Schematic diagram of the cathode-free AEM Zn-Cu pouch cell. (B) Cycling performance and CE of the cathode-free AEM Zn-Cu pouch cell at a current density of 5 mA cm^{-2}. The cathode-free AEM Zn-Cu pouch cell was assembled by replacing the Cu metal cathode with CP. This configuration exhibited a stable cycling performance and reached a high average CE of 98.4% over 120 cycles, indicating the excellent cycle reversibility of our AEM Zn-Cu pouch cell.

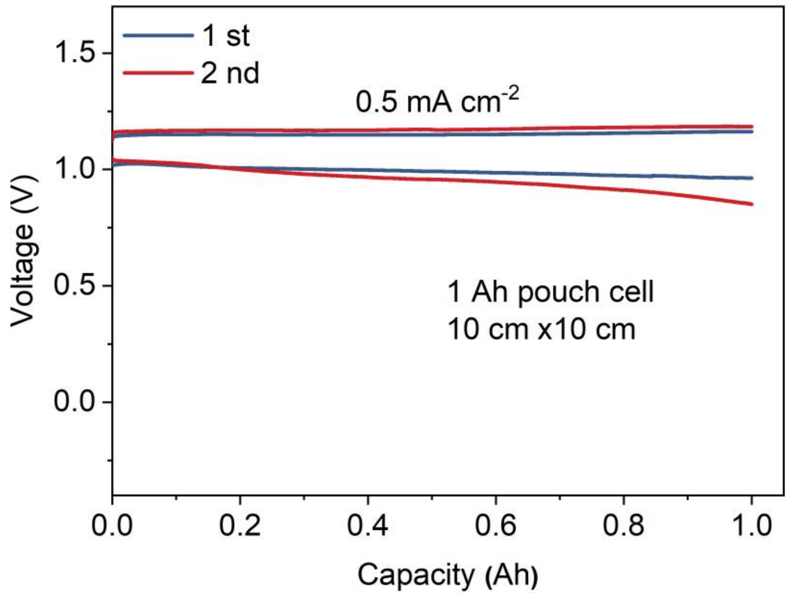

Fig. S39. GCD curves of the AEM Zn-Cu pouch cell with a high capacity of 1 Ah at 0.5 mA cm^{-2}.

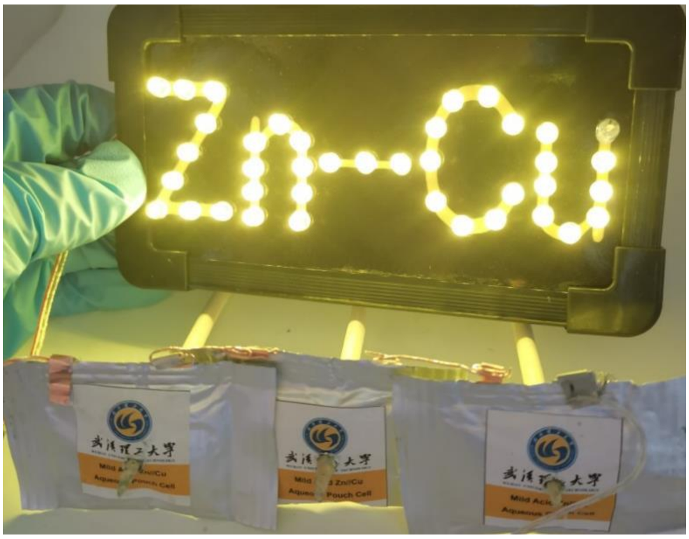

Fig. S40. LEDs lit by a series of three AEM Zn-Cu pouch cells after being punctured.

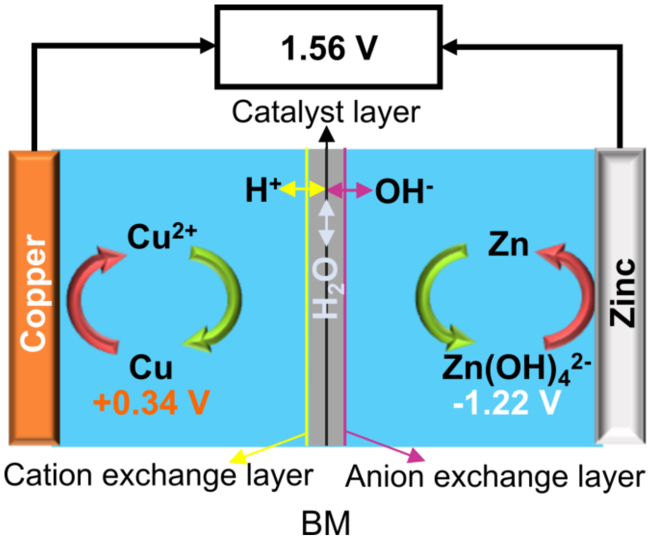

Fig. S41. Reaction mechanism of the alkaline-acid hybrid Zn-Cu battery with the bipolar membrane (BM). The BM consists of an anion exchange layer, a catalyst layer, and a cation exchange layer. H_2O is dissociated into H^+ and OH^- ions on the catalyst layer when exceeding a potential difference of ~0.8 V. During discharging, Zn dissolves on the anode side while Cu deposits on the cathode side, and H^+ and OH^- ions generated from the catalyst layer transfer to catholyte and anolyte, respectively, to compensate for the charge. During charging, Zn plats on the anode side while Cu strips on the cathode side, and H^+ and OH^- ions in catholyte and anolyte, respectively, transport into the membrane to form H_2O.

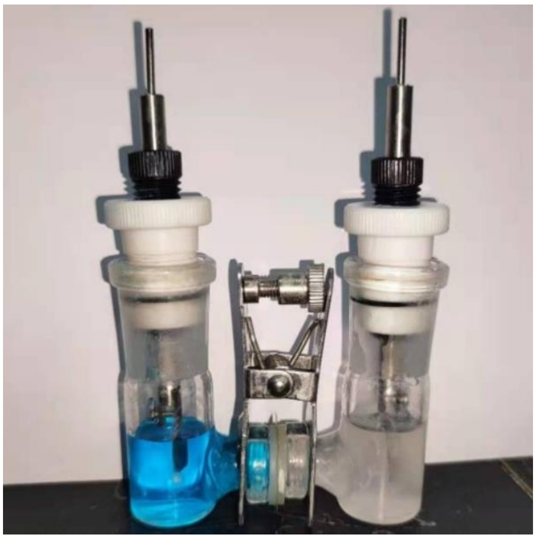

Fig. S42. Digital photo of the alkaline-acid hybrid Zn-Cu mould cell. The left tube contains the Cu metal electrode immersed in 1 M $CuSO_4$ (pH =1), the right tube holds the Sn-coated Zn metal electrode immersed in 6 M KOH + 0.3 M $Zn(Ac)_2$, and the BM is used as the separator.

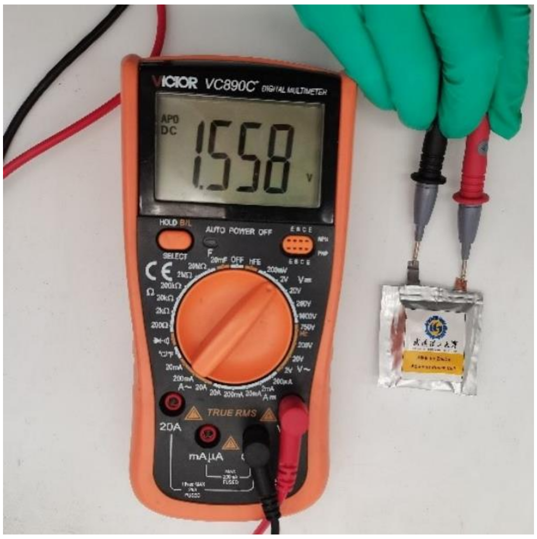

Fig. S43. The open current voltage of the alkaline-acid hybrid Zn-Cu pouch cell.

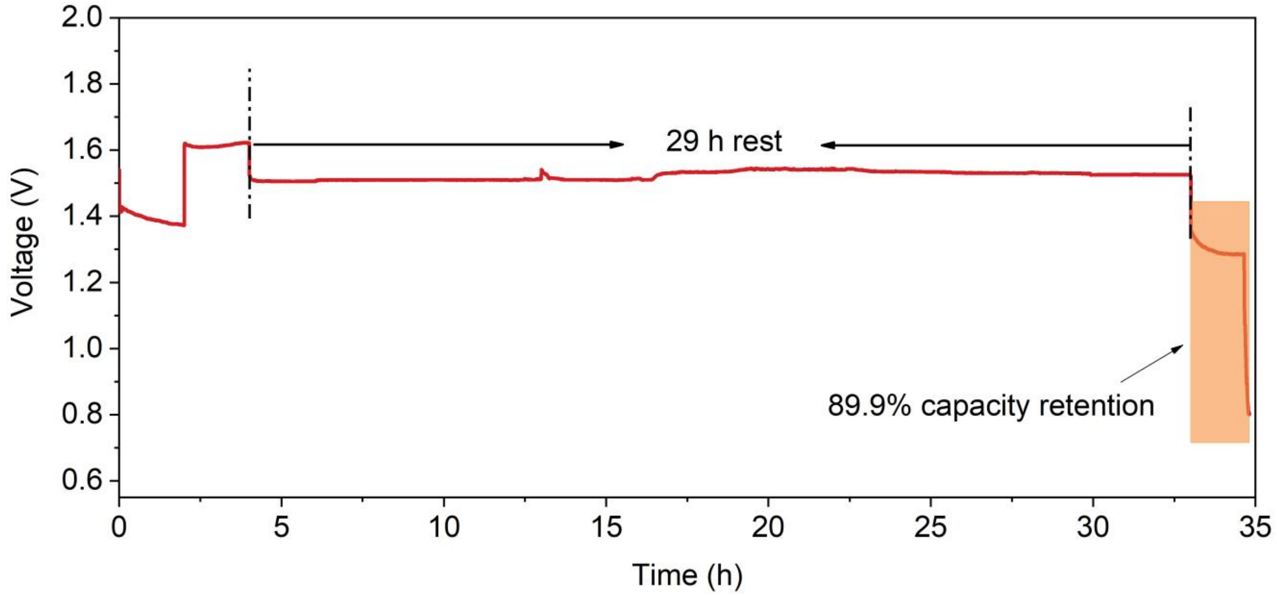

Fig. S44. Self-discharge test of the alkaline-acid hybrid Zn-Cu battery. Firstly, we discharged the alkaline-acid hybrid Zn-Cu cell for 2 h at 1 mA cm^{-2} to provide enough Zn source in the alkaline electrolyte. Subsequently, we replace the Zn metal anode with Sn-coated Ti foil, transitioning to an anode-free cell configuration for the subsequent self-discharge test. The anode free cell was charged for 2 h at 1 mA cm^{-2} and rested for 29 h before the next discharge. The cell shows a discharge capacity of 1.795 mA cm^{-2} after 29 h rest, corresponding to an 89.9% capacity retention.

Table S1. The plating/stripping performance of different metals in aqueous electrolytes

Metal electrode	Redox potential vs SHE (V)	Overpotential (mV)	Cycling performance	ref
Zn	-0.76	~123 (3M $ZnSO_4$, 1 mA cm^{-2}, 0.5 mAh cm^{-2})	400 h	[10]
Fe	-0.44	~400 (0.5 M $FeSO_4$, 0.125 mA cm^{-2}, 0.125 mAh cm^{-2})	500 h	[11]
Mn	-1.18	~90 (1 M $Mn(NO_3)_2$, 1 mA cm^{-2}, 0.08 mAh cm^{-2})	6.7 h	[12]
Al	-1.66	~200 (5 M $Al(OTF)_3$, 0.01 mA cm^{-2}, 0.02 mAh cm^{-2})	200 h	[13]
Cu	+0.34	10 (1 M $CuSO_4$, 0.2 mA cm^{-2}, 0.2 mAh cm^{-2}); 36 (1 M $CuSO_4$, 1 mA cm^{-2}, 1 mAh cm^{-2})	1200 h	This work

Table S2. Parameters of the bipolar membrane[14]

Transmembrane voltage	1.6V① (inorganic salt solution)
	1.25V② (acid and bases)
Hydrolysis efficiency	≥95%
Burst strength	0.25MPa
Thickness	0.20mm
① 1 mol/L NaCl or 0.5 mol/L Na_2SO_4, 100 mA/cm^2, room temperature<br>② 1 mol/L HCl or 1 mol/L NaOH, 100 mA/cm^2, room temperature	

Table S3. Material cost of LIBs and Zn-Cu batteries

	**LIB**	**ZCB**
Positive Electrode, $/kg		
Active material	36.2 (NCM811)<br>33.86 (LCO)<br>10.35 (LFP)	8.4 (Cu)
Carbon	14.46 (SP)	0
Binder	28.58 (PVDF)	0
Binder solvent	2.82 (NMP)	0
Positive current collector, $/kg	2.69 (Al metal) +<br>2.68 (12μ Processing Fee)	0
Negative Electrode, $/kg		
Active material	9.42 (Gr)	3.00 (Zn)
Carbon	14.46 (SP)	0
Binder	14.46 (SBR)+<br>12.29 (CMC)	0
Binder solvent (Water)	0	0
Negative current collector, $/kg	8.4 (Cu metal) +<br>2.75 (8μ Processing Fee)	0
Separator, $/dm²	0.005	31.34 †
Electrolyte, $/kg	12.35 ($LiPF_6$)	2.6 ($CuSO_4$) +<br>0.5 ($ZnSO_4$)† / 0.8 (KOH) †

*Material cost information is mainly obtained from Shanghai Metals Market (SMM) and ICCSINO Database, which were previously reported as data sources for battery material costing[15-17]. Items marked with † are obtained from alibaba.com. The data was retrieved in April 2023.

3. References

1. M. Segall, P. J. Lindan, M. a. Probert, C. J. Pickard, P. J. Hasnip, S. Clark and M. Payne, *Journal of physics: condensed matter*, 2002, **14**, 2717.
2. Q. Li, A. Chen, D. Wang, Z. Pei and C. Zhi, *Joule*, 2022, **6**, 273-279.
3. Y. Deng, A. D. Handoko, Y. Du, S. Xi and B. S. Yeo, *ACS Catalysis*, 2016, **6**, 2473-2481.
4. W. Zhang, C. Huang, Q. Xiao, L. Yu, L. Shuai, P. An, J. Zhang, M. Qiu, Z. Ren and Y. Yu, *J Am Chem Soc*, 2020, **142**, 11417-11427.
5. J. Hao, X. Li, X. Zeng, D. Li, J. Mao and Z. Guo, *Energy & Environmental Science*, 2020, **13**, 3917-3949.
6. X. Guo, H.-S. Xiao, F. Wang and Y.-H. Zhang, *The Journal of Physical Chemistry A*, 2010, **114**, 6480-6486.
7. J. Hao, X. Li, S. Zhang, F. Yang, X. Zeng, S. Zhang, G. Bo, C. Wang and Z. Guo, *Advanced Functional Materials*, 2020, **30**.
8. P. Xiong, Y. Kang, H. Yuan, Q. Liu, S. H. Baek, J. M. Park, Q. Dou, X. Han, W.-S. Jang, S. J. Kwon, Y.-M. Kim, W. Li and H. S. Park, *Applied Physics Reviews*, 2022, **9**.
9. L. Wang, W. Huang, W. Guo, Z. H. Guo, C. Chang, L. Gao and X. Pu, *Advanced Functional Materials*, 2021, **32**.
10. X. Zeng, J. Mao, J. Hao, J. Liu, S. Liu, Z. Wang, Y. Wang, S. Zhang, T. Zheng, J. Liu, P. Rao and Z. Guo, *Adv Mater*, 2021, **33**, e2007416.
11. X. Wu, A. Markir, Y. Xu, C. Zhang, D. P. Leonard, W. Shin and X. Ji, *Advanced Functional Materials*, 2019, **29**.
12. S. Bi, S. Wang, F. Yue, Z. Tie and Z. Niu, *Nat Commun*, 2021, **12**, 6991.
13. C. Wu, S. Gu, Q. Zhang, Y. Bai, M. Li, Y. Yuan, H. Wang, X. Liu, Y. Yuan, N. Zhu, F. Wu, H. Li, L. Gu and J. Lu, *Nat Commun*, 2019, **10**, 73.
14. Y. Dai, J. Li, L. Chen, K. Le, Z. Cai, Q. An, L. Zhang and L. Mai, *ACS Energy Letters*, 2021, **6**, 684-686.
15. X. Yao, E. Olsson, J. Zhao, W. Feng, W. Luo, S. Tan, M. Huang, Y. Zhao, J. Huang and Q. Cai, *Journal of Materials Chemistry A*, 2022, **10**, 2917-2923.
16. Y. Tian, G. Zeng, A. Rutt, T. Shi, H. Kim, J. Wang, J. Koettgen, Y. Sun, B. Ouyang and T. Chen, *Chemical reviews*, 2020, **121**, 1623-1669.
17. X. Sun, M. Ouyang and H. Hao, *Joule*, 2022, **6**, 1738-1742.
